# Distinct regional patterns of synaptic vulnerability across hippocampal and parahippocampal subregions in Alzheimer's disease

**DOI:** 10.1111/bpa.70081

**Published:** 2026-02-09

**Authors:** Maud M. A. Bouwman, Irene Frigerio, Yvon Galis‐de Graaf, Wilma D. J. van de Berg, Laura E. Jonkman

**Affiliations:** ^1^ Department of Anatomy and Neurosciences, Section Clinical Neuroanatomy and Biobanking Amsterdam UMC, Vrije University Amsterdam Amsterdam The Netherlands; ^2^ Amsterdam Neuroscience, Program Neurodegeneration Amsterdam The Netherlands; ^3^ Amsterdam Neuroscience, Program Brain Imaging Amsterdam The Netherlands

**Keywords:** Alzheimer's disease, hippocampal subfields, histopathology, parahippocampal cortex, synapses, synaptophysin

## Abstract

Synaptic loss in the (para)hippocampus is a major contributor to cognitive decline in Alzheimer's disease (AD), yet its regional specificity and pathological correlates remain poorly understood. Here, we quantified synaptophysin‐positive puncta across hippocampal subregions (CA4, CA2, CA1, and subiculum) and parahippocampal cortex (entorhinal cortex, parahippocampal and fusiform gyrus) in postmortem tissue from 28 AD and 17 controls, and assessed relationships to neuropathological severity and cognitive decline. Amyloid‐β, phosphorylated tau (p‐tau) load and neurofilament light (NfL) immunoreactivity were quantified, and Clinical Dementia Rating scores were collected as a cognitive measure. Group differences were analyzed with linear mixed models; correlations between synaptic puncta, pathology and cognitive scores with linear regressions, corrected for age, sex and multiple comparisons. We found selective synaptic loss in the entorhinal cortex (−14%, *p* = 0.017) and parahippocampal gyrus (−14%, *p =* 0.021) in AD versus controls. Hippocampal synaptic density negatively correlated with amyloid‐β in controls (*r* = −0.52, *p* < 0.001) and positively in AD (*r* = 0.25, *p* = 0.007), suggesting a disease‐dependent shift. In AD, hippocampal, but not parahippocampal, subregions with higher p‐tau burden showed greater synaptic density (*r* = 0.21, *p* = 0.003), raising questions about the role of p‐tau in synaptic loss at late stages. Axonal damage (i.e., higher NfL load) in the parahippocampal cortex associated with synaptic loss locally and in interconnected subregions. Worse cognitive performance was strongly associated with synaptic loss in the CA1 (*r* = −0.64, *p* = 0.003), subiculum (*r* = −0.62, *p* = 0.012), entorhinal cortex (*r* = −0.60, *p* = 0.008), parahippocampal (*r* = −0.48, *p* = 0.041) and fusiform gyrus (*r* = −0.67, *p* = 0.002). These findings highlight the vulnerability of the entorhinal cortex and parahippocampal gyrus to synaptic loss in AD. Our results suggest that amyloid‐β and p‐tau pathology may play a limited role in synaptic loss at end‐stage disease, and the strong link between synaptic loss in (para)hippocampal subregions and cognitive decline underscores the need for monitoring synaptic change in light of disease progression and evaluating therapeutic interventions.

## Graphical abstract

Synaptic loss in Alzheimer's disease predominantly affects the entorhinal cortex and parahippocampal gyrus. Amyloid‐β and p‐tau pathology show global associations with synaptic density but are limited in specific subregions. Instead, axonal damage associates with synaptic loss locally and in interconnected subregions. Worse cognitive performance strongly associates with selective subregional synaptic loss.

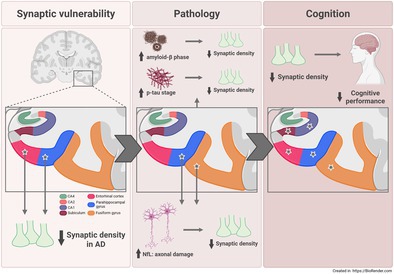

## 1 INTRODUCTION

Synaptic loss is consistently found in Alzheimer's disease (AD) post‐mortem brain tissue and is considered a key pathological process that precedes neuronal death and brain atrophy [1, 2, 3, 4, 5, 6, 7]. Most extensive synapse loss is observed in the hippocampus, as shown in post‐mortem human AD tissue utilizing methods such as unbiased stereological sampling coupled with electron microscopy [8, 9], synaptophysin immunoblotting [10], synaptophysin(‐like) [11, 12] or synaptic vesicle 2A protein (SV2A) [12] immunoreactivity, and human in vivo SV2A PET‐tracer UCB‐J studies [13, 14, 15, 16]. These changes in synaptic density in the hippocampus and association cortices, including the parahippocampal cortex, are the strongest correlate of cognitive decline in patients with AD [1, 2]. Hippocampal synaptic dysfunction and loss can disrupt signal transduction, leading to corticocortical connectivity failure and ultimately cognitive impairment [17, 18]. Most studies focused on synapse loss in the hippocampus as a whole [11, 13, 14], despite the hippocampus being a heterogeneous structure composed of different subfields that may vary in their vulnerability to synaptic loss and their impact on cognitive function [8, 9, 12].

It is believed that pathogenic amyloid‐β and phosphorylated tau (p‐tau) proteins drive synaptic deficits in AD via interactions with synaptic proteins leading to synapse elimination, followed by axonal degeneration, and ultimately neuronal loss [19]. But the spatial relationship between protein aggregation and synaptic loss remains unclear. Studies found synaptic loss to be greater near amyloid‐β plaques [20, 21], with amyloid‐β exerting direct toxic effects at the synapse [22]. Moreover, already at early AD stages, intracellular oligomeric tau protein interacts directly with synaptic vesicles [23] and accumulates at the presynaptic terminal [24], affecting synaptic functioning and eventually leading to synaptic loss [25]. Investigating the regional association between the pathological amyloid‐β and p‐tau burden and synaptic loss could provide valuable insights into the disturbance of neuronal circuits and cognitive deficits.

Axonal damage is another key pathological feature of AD that may contribute to synaptic degeneration [26, 27]. The hippocampus and parahippocampal cortex are highly vulnerable to axonal damage, disrupting normal axonal transport and affecting synaptic plasticity [19, 28]. Given the close interplay between axonal integrity and synaptic function, axonal damage may further exacerbate synaptic degeneration [27].

Synaptic integrity in hippocampal and parahippocampal subregions may be affected in a region‐specific manner in AD, potentially impacting cognitive function. Here, we aim to characterize synaptic loss in hippocampal and parahippocampal subregions in AD and controls brain donors and investigate its association with the severity of amyloid‐β and p‐tau accumulation, axonal damage, and cognitive decline using a high‐resolution histological approach. The findings of this study could enhance our understanding of regional vulnerability to synaptic degeneration and improve interpretation of synaptic change in in vivo studies.

## 2 MATERIALS AND METHODS

### 2.1 Donor inclusion

**FIGURE 1:**
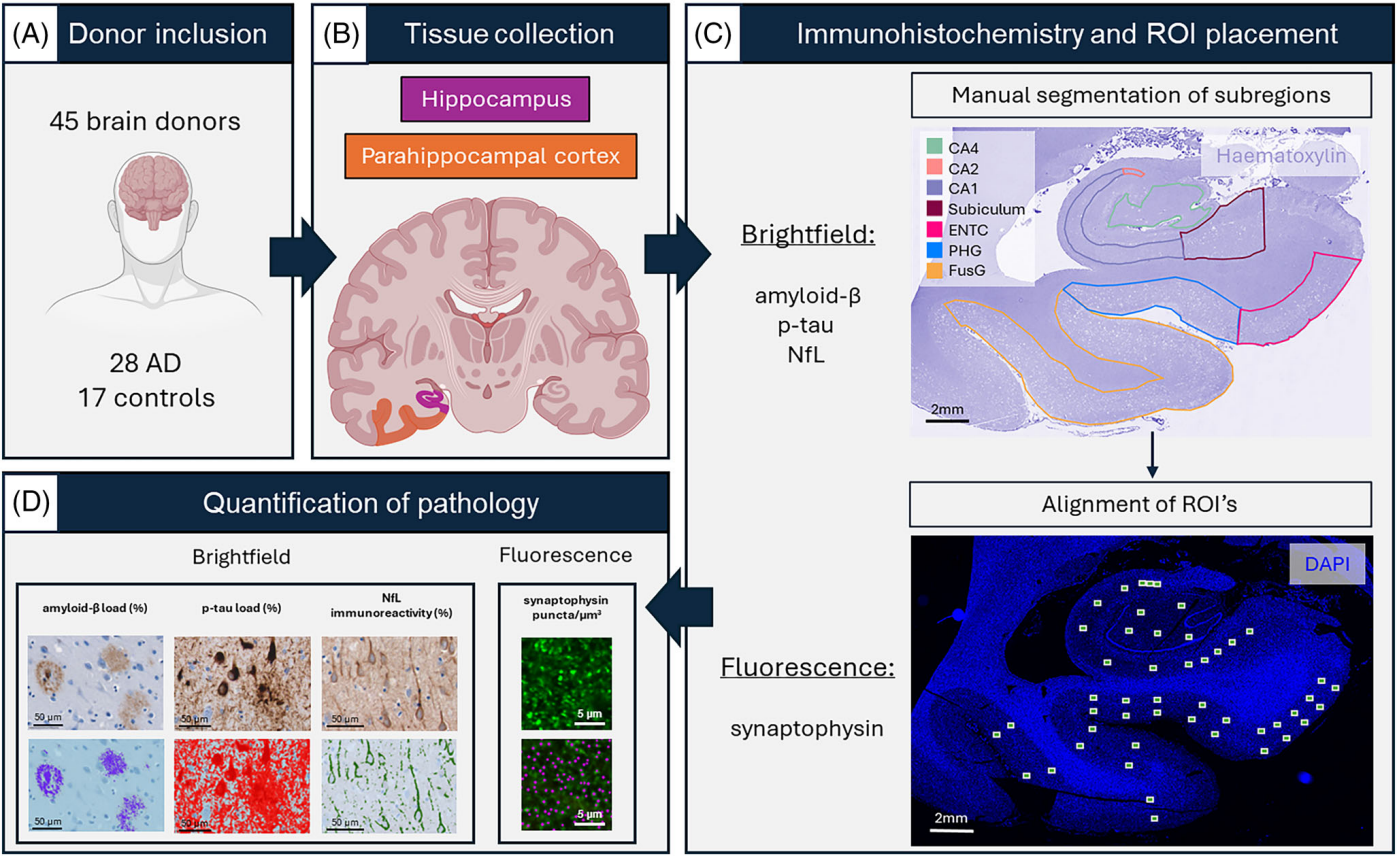
Methods workflow. (A) In total 28 AD and 17 controls brain donors were included; brain collection and dissection were performed and (B) hippocampal (purple) and parahippocampal cortex (orange, comprising entorhinal cortex, parahippocampal and fusiform gyrus) tissue was collected from the right hemisphere. (C) The tissue was formalin‐fixed and paraffin embedded, and processed for immunohistochemistry using antibodies targeting amyloid‐β, p‐tau, NfL with chromogens, and synaptophysin with fluorophores. Whole tissue slides were digitalized and the subregions within the hippocampus and parahippocampal cortex were manually segmented using the haematoxylin counterstain channel delineating the CA4, CA2, CA1, subiculum, entorhinal cortex, parahippocampal and fusiform gyrus, as previously described in Bouwman et al. [41]. Corresponding regions of interest (ROIs) (200 × 160 μm) were placed in the DAPI channel of the fluorescent images. For the parahippocampal cortex, the superficial (layers I–III) and deep (layers IV–VI) cortical layers were taken in account. (D) Subsequently, pixel classifiers were used in QuPath to quantify amyloid‐β and p‐tau load and NfL immunoreactivity on brightfield images. The ROIs placed on fluorescent images were preprocessed, as previously described in Frigerio et al. [39] and synaptophysin^+^ synaptic puncta were quantified per μm^3^ and corrected for cortical thickness as described in Figure S1. AD, Alzheimer's disease; CA, Cornu Ammonis; ENTC, entorhinal cortex; FusG, fusiform gyrus; NfL, neurofilament light chain; p‐tau, phosphorylated tau; PHG, parahippocampal gyrus; ROI, region of interest.

A total of 45 brain donors (28 AD and 17 controls) were included via the Normal Aging Brain Collection Amsterdam (NABCA; http://nabca.eu) [29] and the Netherlands Brain Bank (NBB; http://brainbank.nl). The control donors had no reported history of neurological illnesses. Within the AD group, amnestic (i.e., typical AD, *N* = 13) and non‐amnestic (i.e., atypical AD, *N* = 14) clinical phenotypes were identified. [30] One AD donor could not be classified based on clinical presentation. Furthermore, 17 donors could be considered as early‐onset AD (EOAD) (i.e., clinical diagnosis <65 years) and 11 donors as late‐onset AD (LOAD) (i.e., clinical diagnosis ≥65 years) [31]. The clinical diagnosis was neuropathologically confirmed by an expert neuropathologist according to the international guidelines of the Brain Net Europe II (BNE) consortium (https://www.brainbank.nl/about-us/brain-net-europe/). Individual donor characteristics are listed in Table S1. From neuropathological diagnosis, Thal phase [32], Braak neurofibrillary tangles (NFT) [33] and Braak α‐synuclein [34] stages, as well as the presence of cerebral amyloid angiopathy (CAA) [35] and limbic‐predominant age‐related TDP‐43 encephalopathy (LATE) [36] were collected. The following inclusion criteria from the neuropathological report were considered for controls: Thal phase ≤2 and Braak NFT stage ≤1, to minimize the effect of neuropathology on synaptic density in the (para)hippocampal regions. AD donors were included based on the AD neuropathologic change score of ‘intermediate’ or ‘high’ [37]. In addition, for both controls and AD donors, Braak α‐synuclein stage >2 was used as an exclusion criterion, to avoid the presence of α‐synuclein pathology in the hippocampus. Of note, one AD donor with a Braak α‐synuclein stage of 3 was included, given that α‐synuclein pathology was restricted to the substantia nigra and absent from the hippocampus. Age at diagnosis, disease duration (age at death minus age at diagnosis) and Clinical Dementia Rating (CDR) scores [38], as a measure for overall cognition, were collected from clinical files of the AD donors and reported when available. All donors signed a written informed consent for brain donation and the use of their brain tissue and medical records for research purposes. The workflow is summarized in Figure 1.

### 2.2 Hippocampus tissue sampling and immunohistochemistry

Hippocampus medius tissue from the right hemisphere was collected at brain autopsy and fixated in 4% buffered formalin (pH 7.4). After paraffin embedding, 6‐μm‐thick sections were cut using a microtome for immunohistochemical analysis. All sections were stained for synaptophysin (C‐terminal, clone DAK‐SYNAP), amyloid‐β (clone 4G8), p‐tau (clone AT8) and NfL (amino acid sequence 1–376), as previously described by Frigerio et al. [39]. For detailed information on primary and secondary antibodies see Table S2. Briefly, all sections were deparaffinized, followed by antigen retrieval in a steam cooker for 30 min. The sections were blocked for endogenous peroxidase with 1% hydrogen peroxide in tris buffered saline (TBS; pH 7.4) and consequently with 3% normal donkey serum in TBS (Triton 0.5%). Primary antibodies were added to the sections, diluted in 1% normal donkey serum in TBS (Triton 0.1%), and incubated at 4°C overnight. For the synaptophysin staining, Triton was omitted to avoid synaptic vesicles to be washed away. Moreover, primary antibodies against synaptophysin were detected with donkey anti‐mouse Alexa 488. Sections were counterstained with DAPI and mounted with Mowiol (Sigma‐Aldrich, St. Louis, United States) containing 2.5% 1,4‐diazobicyclo‐[2.2.2]‐octane (DABCO 33‐LV, #290734, Sigma‐Aldrich). For brightfield stainings (amyloid‐β, p‐tau and NfL), the primary antibodies were detected using EnVision (Dako, Glostrup, Denmark), and visualized using 3.3′‐Diaminobenzidine (DAB, Dako) with Imidazole in Tris–HCl (pH 7.6). The sections were counterstained with haematoxylin, dehydrated and mounted with Entellan (Merck, Darmstadt, Germany).

### 2.3 Image analysis

#### 2.3.1 3D synaptic puncta quantification

Imaging of synaptic puncta was randomized and performed using a whole‐slide scanner (Olympus VS200 Evident). First, an overview scan was made of the sections in the DAPI channel at 100× magnification. Based on this scan, regions of interests (ROIs) (200 × 160 μm) were placed within the corresponding brightfield (para)hippocampal segmentations to ensure correspondence between ROIs from pathological/neurofilament and synaptic measurements. The number of ROIs assigned to each region was determined based on the size of each (para)hippocampal subfield: three ROIs in the CA2, five ROIs in the CA4, CA1 and subiculum, 10 ROIs in the entorhinal cortex, parahippocampal and fusiform gyrus. Specifically, for the parahippocampal cortex, five ROIs were placed in cortical layer III and five ROIs in cortical layers V–VI, corresponding to superficial and deep pyramidal layers. The ROIs were scanned with a 60× oil objective on three channels: DAPI, Alexa 488 and autofluorescence (see Table S3 for scanning details). Images were taken as z‐stacks (5 steps, step size of 0.28 μm). Artifacts in the scans were assessed through visual inspection, and ROIs were re‐scanned if necessary. Image processing was previously described by Frigerio et al. [39]. Briefly, autofluorescence signal was subtracted from the images by cross‐talk correction, and images were deconvoluted in Huygens Professional (https://svi.nl/Huygens-Professional). To quantify the synaptic density over volume, neuropil volume was defined by applying inverted masks for nuclei, autofluorescent features, holes and tissue artifacts (such as folds and bubbles) using NIS‐elements (https://www.microscope.healthcare.nikon.com/products/software/nis-elements). Synaptic puncta were quantified with the ‘bright spots’ function, based on diameter (0.6 μm) and intensity. The outcome measure was synaptophysin^+^ puncta/μm^3^ per ROI, yielding a total of ~2000 data points across all cases and regions. Extreme outliers (>3 times the interquartile range) were excluded.

As atrophy of brain tissue is common in AD, synapses may become more tightly packed together, which can result in an artificially increased measurement of synaptic density. Therefore, each data point in AD cases was adjusted for the ratio of corresponding (allo)cortical thickness in AD and controls, as previously applied by Bavarsad et al. [12]. The following formula was used to normalize the synaptic puncta in each subregion of AD donors: normalized synaptic puncta = synaptic puncta × (mean cortical thickness subregion in AD/mean cortical thickness subregion in controls). Cortical thickness was measured in adjacent brightfield stained sections. In the parahippocampal cortex (i.e., entorhinal cortex, parahippocampal gyrus and fusiform gyrus), cortical thickness was measured by calculating the thickness perpendicular to the gray/white matter border (e.g., see Figure S1). The following guidelines were applied to measure hippocampal subfield thickness: (i) The thickness of the subiculum was measured at the border of the inferior horn of the lateral ventricle, avoiding turns wherever possible. (ii) The thickness of the CA1 was measured at three distinct points to account for positional variability: one at the subiculum‐CA1 border, one approximately at the midpoint and one at the CA1–CA2 border. The average of these three measurements was used to calculate the thickness ratio. (iii) The thickness of the CA2 was measured perpendicularly to its outer borders. (iv) For the CA4, no measurements were taken: the shape, and therefore the thickness, varied greatly depending on the cutting level, making the correction unreliable. Average thickness values for the different subregions in controls and AD are presented in Table S4.

#### 2.3.2 Amyloid‐β, p‐tau and NfL quantification

Immunostained hippocampal sections were scanned using a Vectra Polaris at 200× magnification and quantified using QuPath 0.2.3 (https://qupath.readthedocs.io/en/0.2/) [40]. Hippocampal sections were manually segmented into subfields (i.e., CA4, CA2, CA1 and subiculum) and adjacent parahippocampal cortex (i.e., entorhinal cortex, parahippocampal and fusiform gyrus). Segmentations were made in the haematoxylin channel, blinded to the pathology, based on cytoarchitectural boundaries as previously described in Bouwman et al. [41]. Based on the distinct regional vulnerability to neuropathological burden within the hippocampus [41], we selected the CA1 and subiculum as subregions expected to show pronounced pathology, and the CA4 and CA2 as relatively spared regions. This thereby allowed us to assess subregional differences in synaptic vulnerability, while providing an overview of synaptic pathology of the whole hippocampus. Additionally, the parahippocampal cortex was segmented into superficial (layers I–III) and deep (layers IV–VI) cortical layers, which are expected to be affected differently by pathology [42, 43, 44]. Note that the deep layers of the entorhinal cortex consisted of the lamina dissecans plus layers V–VI [45]. DAB immunoreactivity was quantified with previously published *in‐house* QuPath scripts [46, 47]. Pixel classifiers were used to measure the %area load for amyloid‐β, p‐tau and NfL (expressed as %immunoreactivity) for each (para)hippocampal subregion.

### 2.4 Statistical analysis

Statistical analysis was performed using IBM SPSS 28.0 (Chicago, IL). All variables were tested for normality. Demographics between controls and AD were compared using one‐way ANOVA for continuous data and a Chi‐square test of categorical variables. A logarithmic transformation (log_10_) was applied to synaptic density measures to approximate a normal distribution. Linear mixed models were used to compare the nested synaptic data (seven (sub)regions per donor and 3–10 ROIs per (sub)region) between controls and AD, with case ID and (para)hippocampal region as nested variables and age and sex as covariates. Averaged synaptic puncta per (sub)region were used to correlate synaptic density to subregional neuropathological amyloid‐β and p‐tau load, NfL immunoreactivity and cognitive decline, using partial correlations with age and sex as covariates. Control and AD donors were analyzed separately for amyloid‐β and p‐tau, as the minimal pathological load in controls could introduce a group effect in the association. Linear regression models were used to analyze the associations between synaptic puncta and neuropathological load and NfL immunoreactivity when multiple subregions were grouped together. Post‐hoc corrections were performed using false discovery rate (FDR) [48] method to correct for multiple (sub)regions. FDR‐corrected *p*‐values lower than 0.05 were considered significant. Besides local associations, additional exploratory analysis was performed on projection associations between pathological burden (amyloid‐β, p‐tau and NfL) in projection (output) region and synaptic density in target (input) region with partial correlations with age and sex as covariates. Group differences in synaptic density were expressed as percentages in the figures (%difference = [(absolute difference)/average control] × 100). Correlation coefficients (*r*) of linear regression model associations were calculated with the formula: *r* = (estimate (β) fixed effect × standard deviation fixed effect)/standard deviation dependent variable. As a quantifiable estimate of cognitive impairment, predicted change in CDR (ΔCDR) per 1 SD decrease in synaptic density for each subregion was calculated using the formula: ΔCDR = standardized β (synaptic density) × standard deviation (synaptic density).

### 2.5 Data and code availability

The data that support the finding of this study are available via the corresponding author upon reasonable request. QuPath scripts used for brightfield pathological quantification (amyloid‐β and p‐tau %area load and NfL %immunoreactivity) are openly available at our GitHub repository: https://github.com/NeuroScaleLab/pathological_quantification.

## 3 RESULTS

### 3.1 Cohort description

Clinical and neuropathological data of the AD and control groups are summarized in Table 1. AD and controls did not differ in sex, age at death, and post‐mortem delay (all *p* > 0.05). AD donors had an average age at diagnosis of 60 years (range 32–73) and disease duration of 7 years (range 1–23), with a median CDR of 3 (range 1–3). Around 30% of the control donors were carriers of one APOE ε4 allele, while almost 60% of the AD donors had at least one APOE ε4 allele, but this difference was not significant (*p* = 0.125). Three AD donors were known to carry a PSEN1 mutation and one AD donor carried a TREM2 mutation (donor ID #14, 19, 30 and 31, respectively, in Table S1). Pathologically, AD donors showed significantly higher Thal phase [32] and Braak NFT stage [33] compared to controls (both *p* < 0.001), but not Braak α‐synuclein stage [34] (*p* = 0.813). Additionally, AD donors had a higher prevalence of CAA [35] compared to controls (*p* < 0.001), and AD also showed a greater occurrence of LATE [36] (*p* = 0.034). We analyzed the AD group more in detail, distinguishing clinical phenotype, early‐ from late‐onset AD, and APOE ε4 genotype. Atypical AD had a shorter disease duration compared to typical AD, but furthermore, they showed similar (pathological) characteristics (Table S5). Besides age at diagnosis and death, early‐onset (EOAD; <65 years) and late‐onset (LOAD; ≥65 years) AD did not differ in (pathological) characteristics (Table S6). Except for the APOE ε4 genotype, APOE ε4 carriers and non‐carriers showed no differences in (pathological) characteristics (Table S7).

**TABLE 1:** Cohort demographics.

	Control	AD	*p*‐value
*N*	17	28	
Sex F/M (% F)	8/9 (47%)	9/19 (32%)	0.357
Age at diagnosis, years, mean [range]	–	60 [32–73]	
Disease duration years, mean [range]	–	7 [1–23]	
Age at death, years mean [range]	71 [57–85]	67 [37–84]	0.207
Post‐mortem delay, hours:min, mean [range]	8:34 [4:36–11:25]	7:48 [3:36–11:36]	0.114
CDR, median (*N*) [range]	–	3 (*N*=21) [1–3]	
APOE genotype, *N* (%)			0.125
ε4 non‐carrier	11 (65%)	12 (43%)	
ε4 heterozygous	5 (29%)	12 (43%)	
ε4 homozygous	0 (0%)	4 (14%)	
n.a.	1 (6%)	–	
Pathological characteristics			
Thal phase *N*, 0/1/2/3/4/5	3/8/5/1/0/0	0/0/0/1/2/25	<0.001***
Braak NFT stage *N*, 0/1/2/3/4/5/6	4/9/4/0/0/0/0	0/0/0/0/3/9/16	<0.001***
Braak α‐synuclein stage *N*, 0/1/2/3/4/5/6	15/1/1/0/0/0/0	26/1/0/1/0/0/0	0.813
CAA *N* (%), Absent/type 1/type 2	4 (24%), 13/2/2	27 (96%), 1/19/8	<0.001***
LATE *N* (%), Stage 0/1/2/3	0 (0%), 17/0/0/0	7 (25%), 21/3/2/2	0.034*

*Note*: The number of cases per Thal phase, Braak NFT and α‐synuclein stage is indicates as “stage x/x/x/x/x/x/x”, with each number (“x”) corresponding to the number of cases in that stage. Significant *p*‐values are in bold.

Abbreviations: AD, Alzheimer's disease; CAA, cerebral amyloid angiopathy; CDR, clinical dementia rating; F, female; LATE, limbic‐predominant age‐related TDP‐43 encephalopathy; M, male; NFT, neurofibrillary tangles.

### 3.2 AD donors show lower synaptic density in the entorhinal cortex and parahippocampal gyrus

**FIGURE 2:**
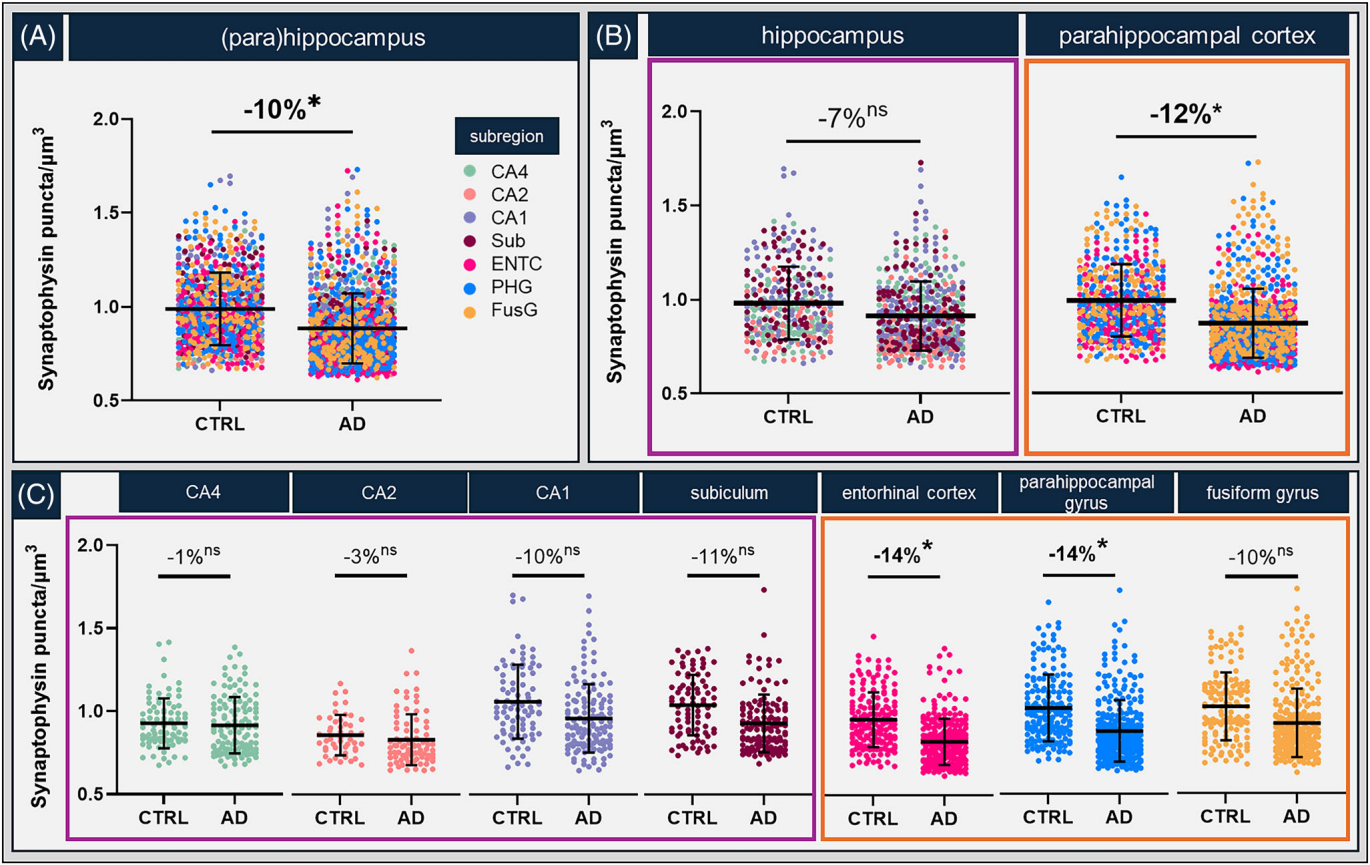
The parahippocampal cortex is vulnerable to synaptic degeneration in AD compared to controls. (A) Group differences between controls and AD in synaptophysin^+^ synaptic density across all (para)hippocampal subregions grouped together, (B) the same datapoints are split in hippocampal (purple outline) or parahippocampal (orange outline) subregion and (C) per individual (para)hippocampal subregion, showing group differences within each brain area. Each datapoint represents one measurement, and multiple measurements per subregion were included for every case. The mean and standard deviation are depicted by the black lines in the graphs. *p < 0.05. AD: Alzheimer's disease; CA: Cornu Ammonis; CTRL: control; ENTC: entorhinal cortex; FusG: fusiform gyrus; ns: not significant; PHG: parahippocampal gyrus; Sub: subiculum.

**FIGURE 3:**
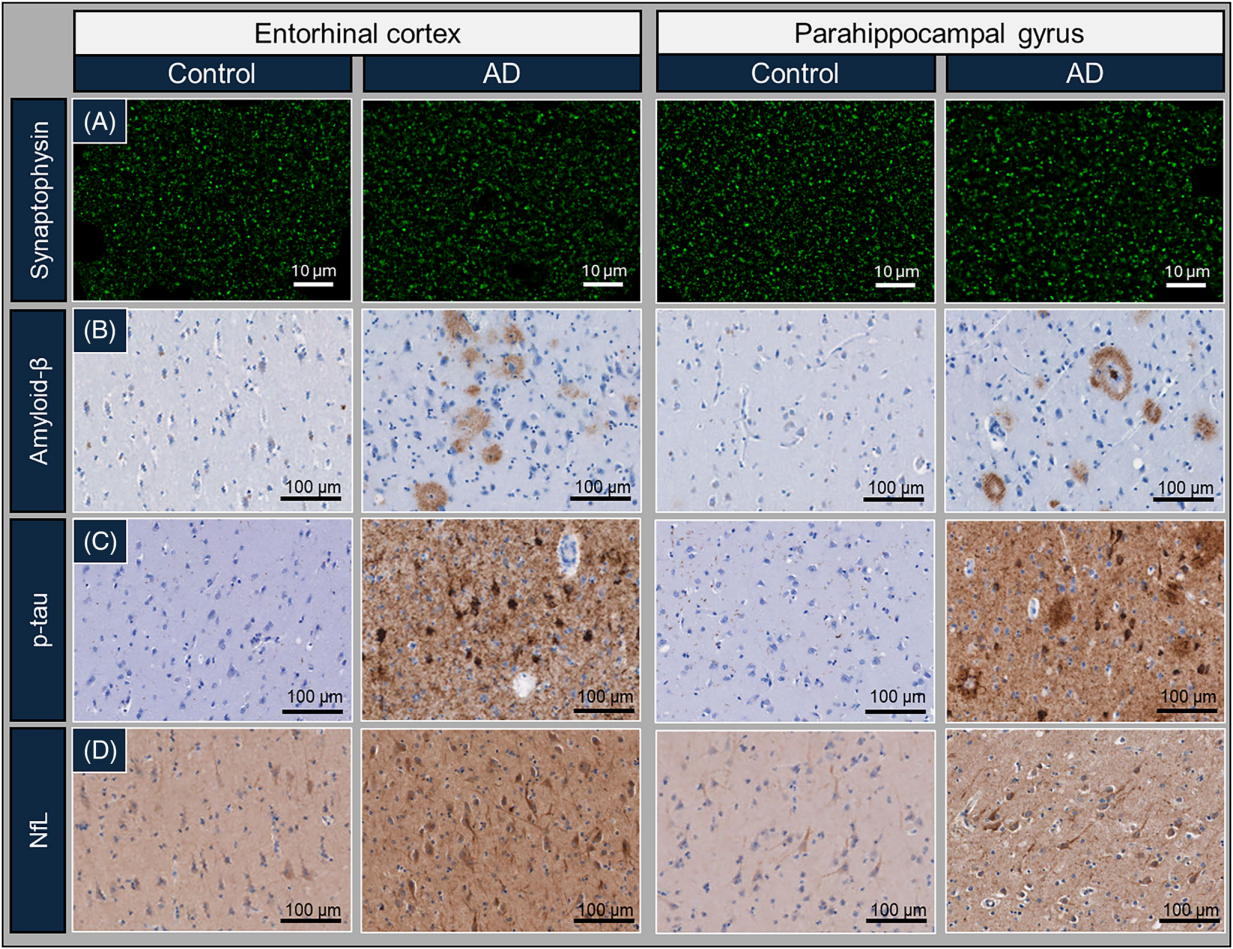
Representative images of synaptic puncta, neuropathological hallmarks, and NfL in controls and AD. Representative images of (A) synaptophysin^+^ pre‐synaptic puncta, (B) amyloid‐β, (C) p‐tau, and (D) NfL were acquired in the superficial layers of the entorhinal cortex and parahippocampal gyrus of control and AD donors. AD: Alzheimer's disease; NfL: Neurofilament light chain; p‐tau: Phosphorylated tau.

Overall, AD donors showed a significant loss of synaptophysin^+^ puncta compared to controls in the (para)hippocampus (−10%, *p* = 0.024) (Figure 2A). Differentiating the parahippocampal cortex (i.e., entorhinal cortex, parahippocampal gyrus and fusiform gyrus combined) from the hippocampus (i.e., CA4, CA2, CA1 and subiculum combined), lower synaptic density was observed in the parahippocampal cortex (−12%, *p* = 0.022) in AD compared to controls, but not in the hippocampus (−7%, *p* = 0.167) (Figure 2B). Within subregions, synaptic density was decreased by 14% in both the entorhinal cortex (*p* = 0.017) and parahippocampal gyrus (*p* = 0.021) in AD compared to controls (Figure 2C). Figure 3 shows representative images of synaptic puncta, amyloid‐β, p‐tau load and NfL in controls and AD of these two regions. Although the other subregions did not show a significant decrease in synaptic density, a modest loss of synapses was still evident in the CA1 (−10%, *p* = 0.338), subiculum (−11%, *p* = 0.110) and fusiform gyrus (−10%, *p* = 0.277), showing high within‐group variability.

We assessed differences in synaptic density between superficial (layer III) and deep (layers V–VI) of the parahippocampal cortex. Synaptic density was higher in the superficial layer compared to deep layers in both controls (+4%, *p* < 0.001) and AD (+3%, *p* = 0.004). Both layers showed loss of synapses in AD compared to controls (superficial: −13%, *p* = 0.013 and deep: −12%, *p* = 0.010), most pronounced in the entorhinal cortex (superficial: −14%, *p* = 0.022 and deep: −14%, *p* = 0.003) and parahippocampal gyrus (superficial: −14%, *p* = 0.010 and deep: −13%, *p* = 0.009, respectively) (Figure S2).

No differences in synaptic density were observed between sexes or AD clinical phenotypes (typical vs. atypical AD). Given the potential modulating effect of early onset disease, CAA and LATE pathology, and genetic predisposition (i.e., APOE ε4 genotype) on synaptic density, subgroup analyses were performed. No subregional differences were observed between subgroups (EOAD vs. LOAD, CAA^+^ vs. CAA^−^, LATE^+^ vs. LATE^−^ and APOE ε4 allele non‐carrier vs. hetero‐ and homozygous carriers) (Figures S3 and S4).

### 3.3 Synaptic loss associates with amyloid‐β, p‐tau, and axonal damage

First, we investigated whether synaptic loss was associated with the spread of amyloid‐β and p‐tau pathology according to Thal phases and Braak stages across the cohort. Synaptic degeneration in the parahippocampal cortex, but not hippocampus, was associated with increasing Thal phase and Braak stage (Thal parahippocampal cortex: *r* = −0.38, *p* = 0.011; Thal hippocampus: *r* = −0.24, *p* = 0.159; Braak parahippocampal cortex: *r* = −0.42, *p* = 0.005; Braak hippocampus: *r* = −0.24, *p* = 0.157) (Figures S5 and S6). Subregional synaptic loss in the subiculum, entorhinal cortex, and parahippocampal gyrus was associated with increasing Thal phase (*r* = −0.38, *p* = 0.040; *r* = −0.45, *p* = 0.009; r = −0.43, *p* = 0.009, respectively). Braak staging seemed to be a bit more sensitive to subregional synaptic loss; besides the subiculum (*r* = −0.40, *p* = 0.020), entorhinal cortex (*r* = −0.48, *p* = 0.001), and parahippocampal gyrus (*r* = −0.47, *p* = 0.003), synaptic degeneration in the CA1 (*r* = −0.36, *p* = 0.042) and fusiform gyrus (*r* = −0.36, *p* = 0.038) was also associated with increasing Braak stage.

Furthermore, we investigated if synaptic loss was associated with local amyloid‐β and p‐tau burden or axonal damage. The distribution of these pathological hallmarks (amyloid‐β, p‐tau and NfL) across (para)hippocampal subregions in controls and AD are depicted in Figure S7. Controls had minimal amyloid‐β and p‐tau pathology, with some diffuse amyloid‐β plaques found in the parahippocampal cortex and mostly neurofibrillary threads (NTs) with some neurofibrillary (pre‐)tangles (NFTs) primarily in the CA1, subiculum, entorhinal cortex and parahippocampal gyrus. In AD, amyloid‐β accumulated primarily in diffuse plaques across the hippocampal subfields, with lake‐like amyloid‐β depositions in the superficial layers of the entorhinal cortex, and the parahippocampal cortex abundant in compact and classic cored plaques, predominantly in the superficial layers. All (para)hippocampal subregions showed abundant NFTs and NTs, with NFTs predominantly observed in the deep layers of the parahippocampal cortex.

**FIGURE 4:**
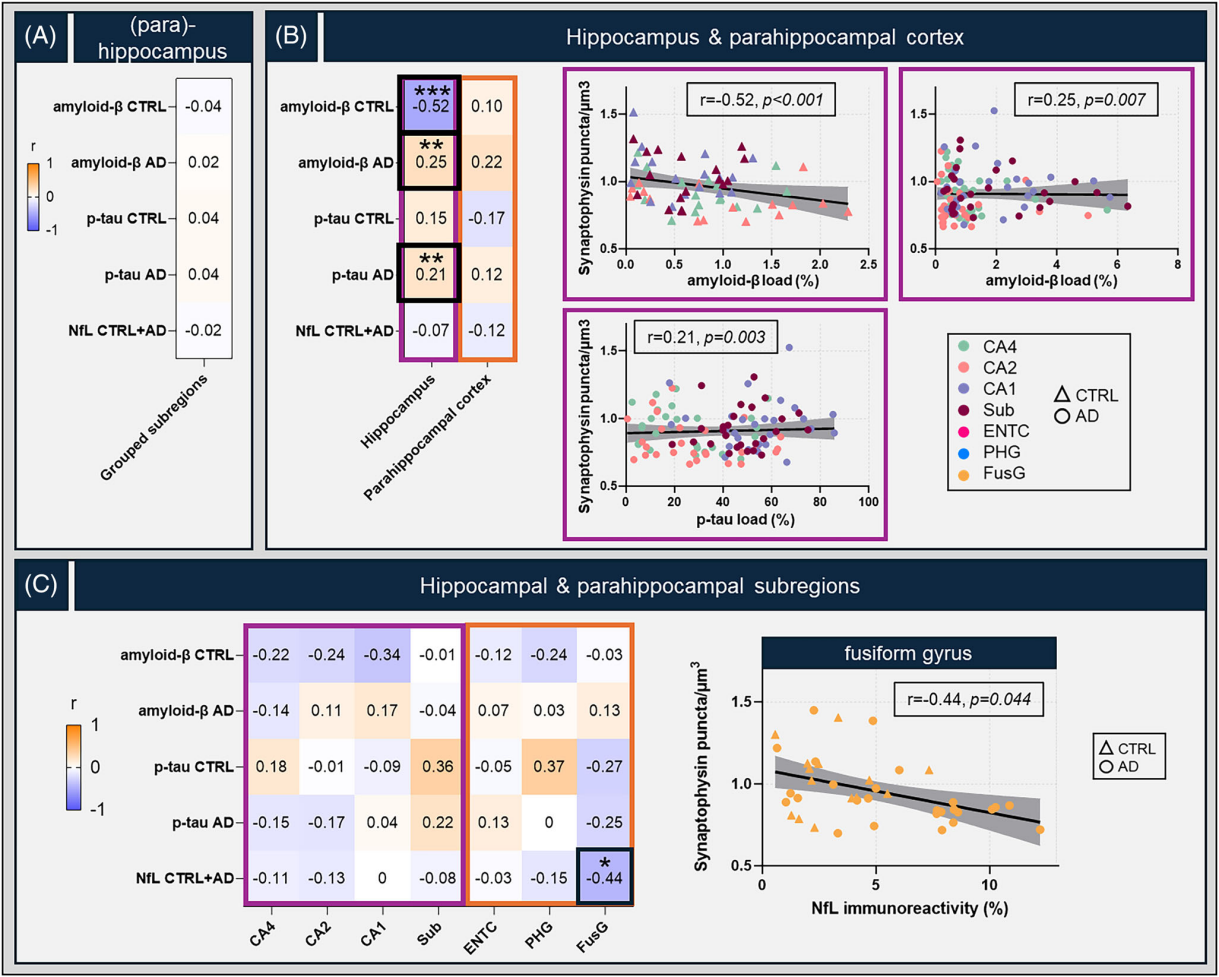
Local associations between synaptic density, neuropathological burden and axonal damage. Correlations between synaptic density and amyloid‐β, p‐tau and NfL are shown for all subregions grouped together (A), hippocampal and parahippocampal cortical subregions grouped together (B) and per individual subregion (C) in heatmaps, color‐coded for correlation coefficient (*r*) with blue representing negative and orange positive correlations. Control and AD donors were analyzed separately for amyloid‐β and p‐tau, as the minimal pathological load in controls would introduce group differences within the associations. Partial correlation *p*‐values were adjusted for multiple comparisons (seven subregions). *p < 0.05, **p < 0.01, ***p < 0.001 after FDR‐correction. Significant correlations, highlighted with a square, are depicted by the scatterplots in which each datapoint represents one averaged measurement, color‐coded for subregion and shape‐coded for group. The purple and orange boxes delineating the heatmaps and scatterplots correspond to the hippocampus and parahippocampal cortex, respectively. AD, Alzheimer's disease; CA, Cornu Ammonis; CTRL, control; ENTC, entorhinal cortex; FusG, fusiform gyrus; NfL, neurofilament light chain; PHG, parahippocampal gyrus; Sub, subiculum.

Control and AD donors were analyzed separately for amyloid‐β and p‐tau, as the minimal pathological load in controls could introduce a group effect in the association. However, since axonal immunoreactivity is observed across both controls and AD without group differences, we combined the two groups for NfL analyses. When all subregions were grouped together, no significant correlations were found between neuropathological markers and synaptic density (Figure 4A).

Control and AD groups showed associations between synaptic density and amyloid‐β load in the hippocampus, but not in the parahippocampal cortex (Figure 4B). The association between amyloid‐β load and synaptic density was negative in controls (*r* = −0.52, *p* < 0.001) while positive in AD (*r* = 0.25, *p* = 0.003), suggesting different mechanisms in physiological aging and AD. Full details of the models can be found in Table S8. Looking at the individual subregions, no significant associations between synaptic density and amyloid‐β burden were found (Figure 4C).

p‐Tau load was positively associated with synaptic density in the hippocampus but not parahippocampal cortex in AD (*r* = 0.21, *p* = 0.003) (Figure 4B). Full details of the models can be found in Table S9. Within subregions, no differences were found between synaptic density and p‐tau burden (Figure 4C). Notably, in AD donors there was a high variability in p‐tau load (Figure S7). When controls and AD were analyzed together—introducing a group effect—all correlations appeared negative (CA4: *r* = −0.16, *p* = 1.000; CA2: *r* = −0.18, *p* = 1.000; CA1: *r* = −0.25, *p* = 0.766; subiculum: *r* = −0.25, *p* = 0.844; entorhinal cortex: *r* = −0.33, *p* = 0.271; parahippocampal gyrus: *r* = −0.42, *p* = 0.044; fusiform gyrus: *r* = −0.38, *p* = 0.115), although only significant in the parahippocampal gyrus (Figure S8).

In the combined control and AD group we found no correlation between NfL immunoreactivity and synaptic density—either when considering the (para)hippocampus as a whole, or within the hippocampus and parahippocampal cortex (Figure 4A,B). Within subregions, axonal damage was associated with synaptic degeneration in the fusiform gyrus (*r* = −0.44, *p* = 0.044; full details of the model can be found in Table S10) (Figure 4C) and a similar association was observed in AD separately (*r* = −0.43, *p* = 0.034), although this did not survive correction for multiple comparisons. Furthermore, in the fusiform gyrus, NfL immunoreactivity was associated with p‐tau load (*r* = 0.50, *p* = 0.009) (Figure S9). For all subregional correlations between neuropathological and axonal markers, see Table S11.

Taken together, we found region‐specific associations between synaptic density and pathological markers, with hippocampal amyloid‐β negatively correlated in controls and positively in AD. Higher hippocampal p‐tau burden seemed to be linked to a higher number of synapses in AD, while axonal damage showed limited local associations, only correlating negatively with synaptic density within the fusiform gyrus.

### 3.4 Pathology associates with synaptic density in interconnected subregions

**FIGURE 5:**
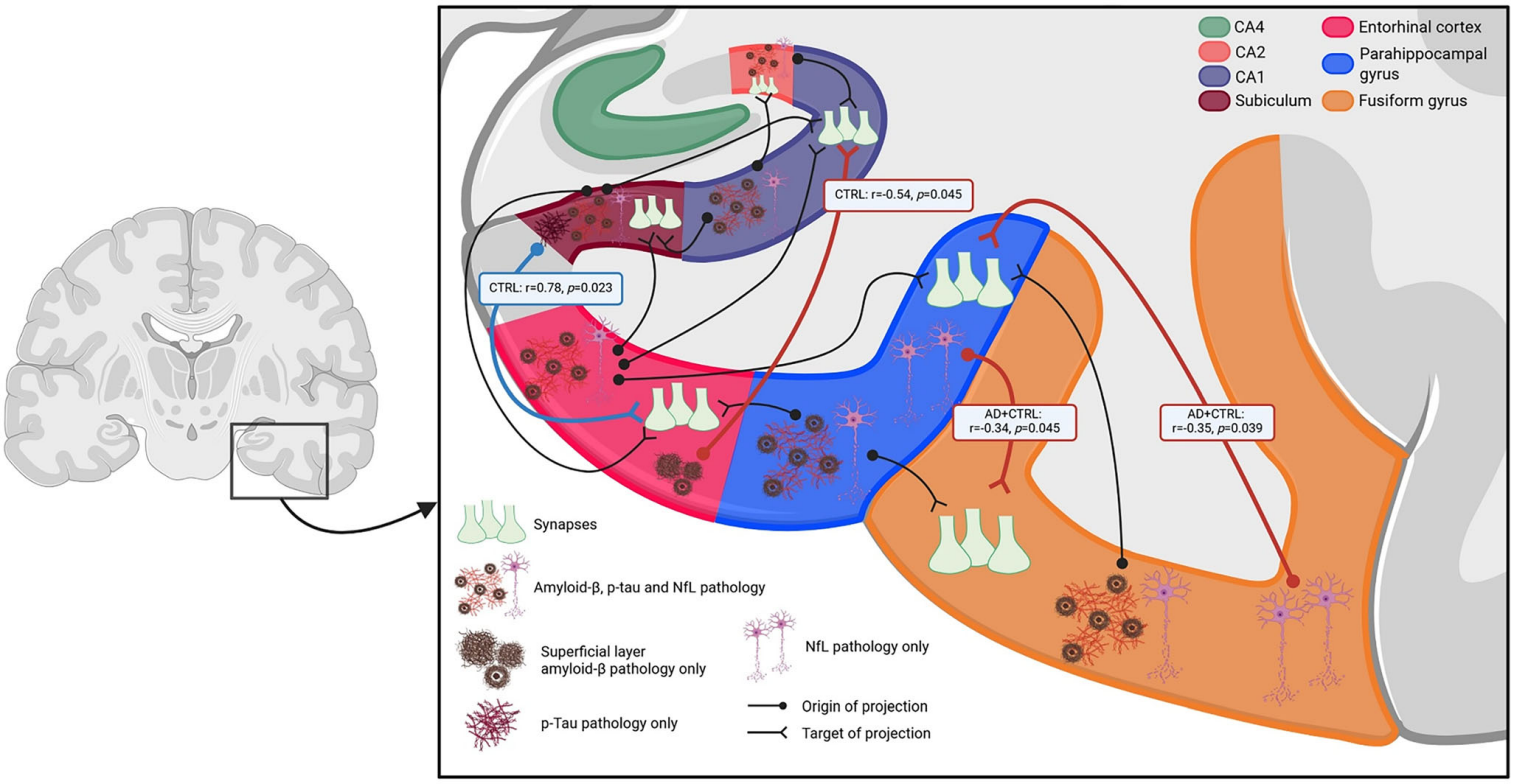
Projecting associations between pathology and synaptic density in interconnected parahippocampal and hippocampal subregions. Here we visualized the projecting connections between the parahippocampal and hippocampal subregions with black projection lines, investigating the impact of pathological accumulation in the projecting region (depicted as a dot) on the synaptic density in the target region (depicted as an open triangle). The blue projection line indicates a significant positive relationship between pathology in the output region and synaptic density in the target region, while red projection lines indicates significant negative relationships. The black projection lines indicated not significant associations. The reported *p*‐values were not corrected for multiple comparisons. Created in https://BioRender.com. AD, Alzheimer's disease; CA, Cornu Ammonis; CTRL, Controls; NfL, neurofilament light chain.

Considering the fact that the (para)hippocampal subregions are highly interconnected [49], we checked whether pathological accumulation in projecting (output) regions was associated with synaptic loss in the target (input) regions (Figure 5). We found that in controls, the minimal p‐tau pathology in the subiculum was associated with an increase in synaptic density in the connecting entorhinal cortex (*r* = 0.78, *p* = 0.023). In the combined control and AD group, higher NfL immunoreactivity in the parahippocampal and fusiform gyri was associated with synaptic degeneration in the targeted fusiform and parahippocampal gyri, respectively (*r* = −0.34, *p* = 0.045 and *r* = −0.35, *p* = 0.039, respectively). No other projecting associations were found between out‐ and input regions (Figure 5). As a further exploration, given that neurons in the superficial layers of the entorhinal cortex specifically project monosynaptically to CA1 [50], we checked whether pathological accumulation in the superficial layers of the entorhinal cortex could be associated with synaptic loss in the CA1. Increased amyloid‐β, but not p‐tau and NfL, load in the superficial layers of the entorhinal cortex was associated with lower CA1 synaptic density in controls only (*r* = −0.54, *p* = 0.045; p‐tau in controls: *r* = 0.37, *p* = 0.192; amyloid‐β in AD: *r* = 0.04, *p* = 0.846; p‐tau in AD: *r* = 0.06, *p* = 0.796; NfL in combined group: *r* = 0.03, *p* = 0.849) (Figure 5). Details on all projection associations can be found in Tables S12–S17.

Overall, axonal damage in the fusiform gyrus was associated not only with local synaptic loss, but also with synaptic loss in the connected parahippocampal gyrus, and vice versa.

### 3.5 Selective (para)hippocampal synaptic loss associates with cognitive decline

Overall synaptic loss correlated with age in controls (*r* = −0.44, *p* = 0.023), but not in AD (*r* = 0.28, *p* = 0.081). Synaptic degeneration was associated with a longer disease duration in AD (*r* = −0.38, *p* = 0.029), while other pathological hallmarks (amyloid‐β, p‐tau, and NfL) did not increase with increasing disease duration (all *p* > 0.543).

**FIGURE 6:**
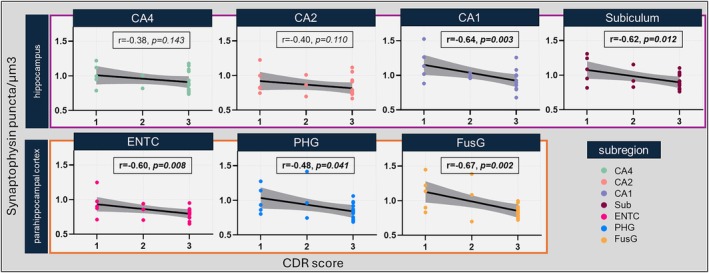
Subregional synaptic loss is associated with cognitive decline in AD. Correlations between synaptic density and CDR scores are shown per subregion. Partial correlation *p*‐values are adjusted for multiple comparisons (seven subregions) with FDR‐correction. Synaptic density and CDR scores significantly correlated in the CA1, subiculum, entorhinal cortex, parahippocampal gyrus, and fusiform gyrus. Each datapoint represents one averaged synaptophysin density measurement per case. *N*_CDR=1_ = 5, *N*_CDR=2_ = 3, *N*_CDR=3_ = 13. From 7 AD cases, no CDR scores were available and therefore missing from this analysis. B/D, behavioural/dysexecutive; CA, Cornu Ammonis; CDR, clinical dementia rating; CTRL, control; ENTC, entorhinal cortex; FusG, fusiform gyrus; LvPPA, logopenic variant of primary progressive aphasia; PCA, posterior cortical atrophy; PHG, parahippocampal gyrus; Sub, subiculum.

In AD, overall synaptic loss correlated with higher CDR scores (*r* = −0.46, *p* = 0.002) in the (para)hippocampus. The same was found for synaptic loss within the hippocampus (*r* = −0.42, *p* = 0.008) and parahippocampal cortex (*r* = −0.52, *p* = 0.002). Within subregions, worse cognitive status associated with synaptic degeneration in the CA1 (*r* = −0.64, *p* = 0.003), subiculum (*r* = −0.62, *p* = 0.012), entorhinal cortex (*r* = −0.60, *p* = 0.008), parahippocampal (*r* = −0.48, *p* = 0.041) and fusiform gyrus (*r* = −0.67, *p* = 0.002) (Figure 6). Full details of the model can be found in Table S18. Synaptic density in the CA4 (*r* = −0.38, *p* = 0.143) and CA2 (*r* = −0.40, *p* = 0.110) subregions did not seem to contribute to cognitive decline. These two subregions also showed the least amyloid‐β and p‐tau pathology in AD (Figure S7). No noticeable differences were observed between the clinical AD phenotypes.

Additionally, we examined the predicted change in CDR (ΔCDR) per 1 standard deviation (SD) decrease in synaptic density for each subregion, as shown in Table S18. These estimates quantify the estimated cognitive decline associated with reductions in synaptic density. The predicted decrease in CDR‐score ranged from 0.022 (CA4) to 0.049 (fusiform gyrus) per 1 SD decrease in synaptic density. While ΔCDR itself does not provide additional insights beyond the correlations presented in Figure 5, it offers a clinically interpretable measure of the expected cognitive impact of synaptic density changes.

Besides synaptic density, we analyzed whether neuropathological load was associated with cognitive scores. Within subregions, amyloid‐β burden did not associate with cognitive status, while higher p‐tau burden in the parahippocampal and fusiform gyrus (*r* = 0.53, *p* = 0.050 and *r* = 0.59, *p* = 0.024), as well as axonal damage in the fusiform gyrus (*r* = 0.61, *p* = 0.043), associated with higher CDR scores.

## 4 DISCUSSION

In this study, we mapped the distribution of synaptic loss across hippocampal subfields and parahippocampal cortex, and assessed whether (sub)regional differences in synaptic density correlate with neuropathological burden, axonal degeneration and cognitive status. We found synaptic loss in the entorhinal cortex and parahippocampal gyrus in AD, but not in the hippocampal subfields. Amyloid‐β negatively correlated with synaptic density in controls but positively in AD. A higher amount of p‐tau pathology seemed to be linked to a higher number of synapses in AD, whereas axonal damage in the fusiform gyrus was associated not only with local synaptic loss, but also with synaptic loss in the connected parahippocampal gyrus, and vice versa. Finally, the loss of synapses in the CA1, subiculum, entorhinal cortex, parahippocampal and fusiform gyrus associated with worse cognitive scores.

Our findings align with previous studies reporting a loss of synaptic density in the parahippocampal cortex in AD [15]. The parahippocampal cortex is responsible for information transfer from the cortex to the hippocampus [51, 52] and is among the earliest and most severely affected regions by AD pathology, including amyloid‐β and p‐tau accumulation [32, 53, 54]. Synaptic degeneration in this region suggests that information flow to the hippocampus is disrupted, altering its synaptic circuitry [51]. Within subregions, we found a 14% reduction in synaptic density in the entorhinal cortex and parahippocampal gyrus in AD compared to controls, suggesting a loss of perforant pathway projections (i.e., from the entorhinal cortex to the hippocampal formation) which are essential for memory formation [55, 56]. Prior post‐mortem and SV2A PET AD studies have reported similar reductions in these subregions [8, 12, 57, 58, 59]. Surprisingly, synaptic density in the hippocampus and CA1, subiculum and the fusiform gyrus was not significantly decreased in our AD donors, despite several reports indicating synaptic loss in these regions [9, 12, 59, 60]. It is important to note that, in contrast to the previous reports, our analysis focused on synaptophysin^+^ puncta, which measures density of pre‐synaptic terminals but does not account potential differences in synaptic types. Synaptic density does not reflect synaptic function; some synapses may be non‐functional, and there could be a compensatory increase in the number of synapses in response to synaptic dysfunction, or loss [18, 61].

In controls, lower hippocampal synaptic density associated with higher amyloid‐β burden, suggesting that diffuse amyloid‐β plaques may contribute to synaptic degeneration as part of physiological aging [62, 63]. Supporting this, we found that age correlated with lower overall synaptic density in controls, but not in AD, underscoring age‐related synaptic degeneration independent of clinical disease. The association between amyloid‐β and synaptic density reversed in AD, where a loss of hippocampal synapses correlated (weakly) with lower amyloid‐β load. Previous studies have found similar results; a PET study by O'Dell et al. reported lower hippocampal synaptic density associated with higher global amyloid‐β deposition in mild cognitive impairment, but with lower global amyloid‐β deposition in AD [64]. The authors suggested that in the early stages of clinical disease, continued amyloid‐β accumulation associates with synaptic degeneration, but at later stages plateaus and becomes uncoupled from neurodegeneration [64], which has been hypothesized before [65]. Meaning, amyloid‐β production is compromised by heavily atrophic hippocampal tissue [64], while synaptic loss continues throughout the course of the disease [66]. This is further supported by our results, which show a decrease in synaptic density, but not an increase in neuropathological load with increasing disease duration.

Another possible explanation for the positive association between amyloid‐β and synaptic density is the space‐occupying nature of compact amyloid‐β plaques [67], therefore pushing surrounding synapses into the nearby neuropil [39], artificially increasing synaptic density [22]. Our group has previously reported a similar positive association between amyloid‐β and synaptic density in Lewy body dementia [39]. Notably, the same analysis is used in the current study, excluding DAPI‐positive amyloid‐β plaques from the neuropil mask, meaning synapses within plaques are not counted and potentially concentrating the number of synapses within a smaller neuropil volume. Moreover, most post‐mortem studies reporting synaptic loss correlations with amyloid‐β levels [68, 69, 70] investigated oligomeric amyloid‐β rather than plaques or load [68, 69]. Overall, future research should investigate the spatial relationship between amyloid‐β plaques and synaptic loss at different (pre)clinical stages of AD.

Even if p‐tau burden is a good correlate of hippocampal atrophy [41, 71, 72, 73, 74], the role of p‐tau in synaptic degeneration remains inconclusive [75]. We observed negative associations between synaptic density and Braak NFT staging, but a positive relationship between p‐tau burden and synaptic density in the hippocampus in AD. These findings suggest that while synaptic loss correlates with the progression of p‐tau pathology, higher p‐tau load in the hippocampus of AD cases may be associated with a relative preservation of synapses. This finding contrasts with previous post‐mortem and PET studies reporting a negative relationship between p‐tau pathology and synaptic density, both in mixed control/AD cohorts [59, 76, 77] and AD separately [77, 78, 79, 80]. Notably, one PET study also reported that while the overall association between tau pathology and synaptic density was negative, AD patients with relatively low tau burden showed a positive association between synaptic density and tau pathology, suggesting possible distinct effects of early versus more advanced stages of tau accumulation. Accumulating evidence furthermore suggests that soluble, rather than aggregated, p‐tau may be synaptotoxic [75, 81, 82]. Alternatively, a compensatory increase in the number of synapses may occur in response to synaptic dysfunction or loss [18, 61]. As disease progresses, the relationship between neuropathology and synaptic density may shift and reach a stage where pathology no longer directly correlates with synaptic degeneration [64, 83]. Additionally, p‐tau is intracellular and highly abundant in atrophic hippocampal subregions at end‐stages of the disease [41], as such the extent of neurodegeneration could lead to underestimation of p‐tau burden [83]. Furthermore, total p‐tau load could also be underestimated as we only detected pre‐ and mature tangles with the AT8 antibody, thereby omitting extracellular ghost tangles [84], which could have confounded the relationship with synaptic density. Together, this suggests a possible non‐linear relationship between p‐tau pathology and synaptic density, indicating that p‐tau accumulation alone may not explain the observed variations in synaptic density.

We found that axonal damage in the fusiform gyrus is associated with a loss of synapses. The fusiform gyrus is crucial for cortical information influx to the hippocampus for memory formation [51, 52], and disruptions in this connection might lead to a decreased cross‐talk between the cortex and hippocampus, which may eventually lead to cognitive impairment [85]. Here, the neurodegenerative processes of synaptic and axonal degeneration might occur together. Axonal transport is essential for maintaining synaptic function, and axonal injury could lead to synaptic loss [28]. Conversely, changes in early local synaptic communication driven by amyloid‐β and p‐tau pathology could disrupt axonal integrity by the dissociation of neurofilaments from microtubules, ultimately leading to axonal damage [62, 86]. Notably, the fusiform gyrus is one of the first regions affected by tau pathology [33]. Here, the strong correlation between p‐tau load and NfL immunoreactivity suggests a region‐specific interplay between p‐tau and axonal damage and may contribute to synaptic dysfunction and degeneration.

As part of the corticohippocampal circuit, hippocampal and parahippocampal subregions are highly interconnected [49, 87]. Pathology impairs signal transmission and may lead to downstream synaptic degeneration in efferent projections [88]. In the present study, synaptic loss in the parahippocampal gyrus in AD was not associated with local amyloid‐β, p‐tau burden or axonal damage, but rather with axonal damage in the afferent projecting fusiform gyrus. This underscores the role of disruptions in axonal transport in communication between interconnected subregions [85]. Additionally, we found that amyloid‐β accumulation in the superficial layers in the entorhinal cortex was associated with synaptic degeneration in its downstream region, the CA1. This suggests that local pathological burden in the entorhinal cortex can influence synaptic integrity in connected hippocampal areas. While this study focused on hippocampal and parahippocampal subregions, it is important to note that hippocampal pathology and synaptic loss likely reflect broader network‐level disruptions, including long‐range projections and cross‐talk with neocortical regions such as the cingulate and prefrontal cortex [89]. These interactions may further modulate hippocampal function and contribute to the observed changes in cognitive decline.

Synaptic loss is widely considered the strongest neuropathological correlate of cognitive decline in AD, even at early disease stages [8, 9, 90, 91, 92]. Our findings confirm that worse cognitive scores are linked to stronger synaptic loss in several hippocampal and parahippocampal subregions, including the CA1, subiculum, entorhinal cortex, parahippocampal and fusiform gyrus. This suggests that synaptic degeneration in these subregions may reflect altered brain connectivity associated with cognitive decline [18]. Synaptic degeneration within individual subregions showed stronger associations with cognitive decline than measures of the whole hippocampus or parahippocampal cortex. This suggests that even subtle synaptic changes in specific subregions (i.e., CA1, subiculum, entorhinal cortex and fusiform gyrus; areas critical for memory processing) may disproportionately impact cognitive function. Notably, synaptic degeneration correlated more closely with cognitive impairment than neuropathological load or axonal damage. Furthermore, lower synaptic density—but not higher neuropathological burden—associated with longer disease duration, consistent with previous findings [66]. These results suggest that synaptic markers may provide a more accurate reflection of cognitive decline and disease progression, highlighting their value as sensitive and clinically meaningful biomarkers.

Our study has several limitations. We used synaptophysin as a pre‐synaptic marker, which is widely used in post‐mortem studies. However, PET studies use SV2A as a marker, making direct clinical translation of our work challenging caused by potential differences in marker specificity [39]. Nonetheless, post‐mortem studies have reported positive correlations between synaptophysin and SV2A measures [12, 39], suggesting some degree of comparability. Additionally, our cohort consisted exclusively of pathologically end‐stage AD donors, which may have led to overestimation of synaptic density caused by severe atrophy. We tried to correct for cortical thinning in our AD group, but we are aware this was only an estimation for the whole region. Our relatively small sample size is another limitation, as it may have reduced statistical power. An increased sample size could also allow for the differentiation of AD clinical phenotypes to determine whether they exhibit distinct patterns of (para)hippocampal synaptic integrity. Lastly, our cognitive analyses rely on global measures (CDR), which reflect overall cognitive function, rather than domain‐specific cognitive function as shown before by Scheff and colleagues [9]. To relate subregional (para)hippocampal synaptic loss to hippocampal‐dependent cognitive function, future studies incorporating domain‐specific neuropsychological tests are needed. Despite these limitations, our high‐resolution 3D histological approach enables detailed mapping of synaptic density distribution across (para)hippocampal subregions and provides valuable insights into its association with neuropathological accumulations and clinical outcomes.

## 5 CONCLUSION

The entorhinal cortex and parahippocampal gyrus are particularly vulnerable to synaptic degeneration in AD. We found a strong association between synaptic loss in (para)hippocampal subfields and cognitive impairment, underscoring the need for monitoring synaptic change in light of disease progression and evaluating therapeutic interventions. Our results suggest that amyloid‐β and p‐tau pathology may play a limited role in synaptic loss at end‐stage disease, although the exact relationship remains to be fully elucidated.

## AUTHOR CONTRIBUTIONS

M.M.A.B. contributed to experimental concept and design, data collection, statistical analysis, interpretation of data, and drafting of the manuscript. I.F. contributed to experimental concept and design, histopathological data collection and interpretation of data. Y.G.G. contributed to data collection. W.D.J.B. performed the NABCA dissections. W.D.J.B. and L.E.J. contributed to the experimental concept and design and interpretation of the data and provided supervision. L.E.J. obtained the project funding. All authors read, edited and approved the final manuscript.

## FUNDING INFORMATION

This study was funded by Alzheimer Nederland (WE.03‐2020‐02) and Stichting LSH‐TKI PPP grant (S‐000438). The authors have no relevant financial or non‐financial interests to disclose.

## CONFLICT OF INTEREST STATEMENT

W.D.J.B. performed contract research for Roche Tissue Diagnostics and Discoveric Bio, received antibodies for research purposes from Hoffmann‐La Roche and Prothena. She is a member of the scientific advisory board of Gain Therapeutics and Alzheimer Netherlands. L.E.J. performed contract research for Imeka.

## ETHICS STATEMENT

All donors signed a written informed consent for brain donation and the use of their brain tissue and medical records for research purposes. The procedures for brain tissue collection of NBB and NABCA have been approved by the Medical Ethical Committee of Amsterdam UMC (formerly known as VU medical center).

## Supporting information


**FIGURE S1:** Example of cortical thickness measurements in brightfield stained (amyloid‐β) section in control and AD.
**FIGURE S2:** Synaptic density differences between controls and AD in layer III and V–VI across parahippocampal subregions.
**FIGURE S3:** Additional analyses in early versus late‐onset Alzheimer's disease brain donors.
**FIGURE S4:** Synaptic density differences between the presence or absence of CAA, LATE and APOE ε4.
**FIGURE S5:** Associations between Thal phase and synaptic density across the cohort.
**FIGURE S6:** Associations between Braak NFT stage and synaptic density across the cohort.
**FIGURE S7:** Distribution of neuropathological load and NfL immunoreactivity across (para)hippocampal subregions in controls and AD.
**FIGURE S8:** Subregional associations between p‐tau load and synaptic density across the cohort.
**FIGURE S9:** Association between p‐tau load and NfL immunoreactivity in the fusiform gyrus in the combined cohort.

**TABLE S1:** Donor characteristics.
**TABLE S2:** Information on primary antibodies.
**TABLE S3:** Information on fluorescence Olympus VS200 scanning at 60× magnification.
**TABLE S4:** Average thickness measurements of (para)hippocampal subregions and ratio used to normalize synaptic puncta in AD donors.
**TABLE S5:** Characteristics of typical versus atypical AD.
**TABLE S6:** Characteristics of early‐onset versus late‐onset AD.
**TABLE S7:** Characteristics of APOE ε4 carrier (+) and non‐carriers (−) AD donors.
**TABLE S8:** Detailed association model between synaptic density and amyloid‐β. *p*‐Values are FDR corrected for multiple subregions. Significant associations are in bold.
**TABLE S9:** Detailed association model between synaptic density and p‐tau load. *p*‐Values are FDR corrected for multiple subregions. As none of the uncorrected *p*‐values were significant in the model of the controls, FDR‐correction was not possible. Significant associations are in bold.
**TABLE S10:** Detailed association model between synaptic density and NfL immunoreactivity. *p*‐Values are FDR corrected for multiple subregions. Significant associations are in bold.
**TABLE S11:** *R*‐values and *p*‐values before and after FDR‐correction of correlations between pathological markers in a combined cohort. As none of the uncorrected *p*‐values were significant for the NfL and amyloid‐β correlation, FDR‐correction was not possible.
**TABLE S12:** Details of projection associations between amyloid‐β and synaptic density in interconnected parahippocampal and hippocampal subregions. As this analysis was exploratory, *p*‐values were not corrected for multiple comparisons.
**TABLE S13:** Details of projection associations between p‐tau and synaptic density in interconnected parahippocampal and hippocampal subregions. As this analysis was exploratory, *p*‐values were not corrected for multiple comparisons.
**TABLE S14:** Details of projection associations between NfL and synaptic density in interconnected parahippocampal and hippocampal subregions. As this analysis was exploratory, *p*‐values were not corrected for multiple comparisons.
**TABLE S15:** Details of projection associations between amyloid‐β in the superficial layers of the entorhinal cortex and synaptic density in interconnected hippocampal subregions. As this analysis was exploratory, *p*‐values were not corrected for multiple comparisons.
**TABLE S16:** Details of projection associations between p‐tau in the superficial layers of the entorhinal cortex and synaptic density in interconnected hippocampal subregions. As this analysis was exploratory, *p*‐values were not corrected for multiple comparisons.
**TABLE S17:** Details of projection associations between NfL the superficial layers of the entorhinal cortex and synaptic density in interconnected hippocampal subregions. As this analysis was exploratory, *p*‐values were not corrected for multiple comparisons.
**TABLE S18:** Detailed association model between synaptic density and cognitive scores. *p*‐Values are FDR corrected for multiple subregions. Significant associations are in bold.

## Data Availability

The data that support the findings of this study are available from the corresponding author upon reasonable request.
