# Supplementary material for: Distinct regional patterns of synaptic vulnerability across hippocampal and parahippocampal subregions in Alzheimer's disease
Source: Brain Pathol. 2026 Feb 9;36(4):e70081. doi: 10.1111/bpa.70081 (PMC13239152; doi:10.1111/bpa.70081)
Supplement: Supplementary file 1 — FIGURE S1: Example of cortical thickness measurements in brightfield stained (amyloid‐β) section in control and AD. FIGURE S2: Synaptic density differences between controls and AD in layer III and V–VI across parahippocampal subregions. FIGURE S3: Additional analyses in early versus late‐onset Alzheimer's disease brain donors. FIGURE S4: Synaptic density differences between the presence or absence of CAA, LATE and APOE ε4. FIGURE S5: Associations between Thal phase and synaptic density across the cohort. FIGURE S6: Associations between Braak NFT stage and synaptic density across the cohort. FIGURE S7: Distribution of neuropathological load and NfL immunoreactivity across (para)hippocampal subregions in controls and AD. FIGURE S8: Subregional associations between p‐tau load and synaptic density across the cohort. FIGURE S9: Association between p‐tau load and NfL immunoreactivity in the fusiform gyrus in the combined cohort. [file BPA-36-e70081-s001.docx]

**
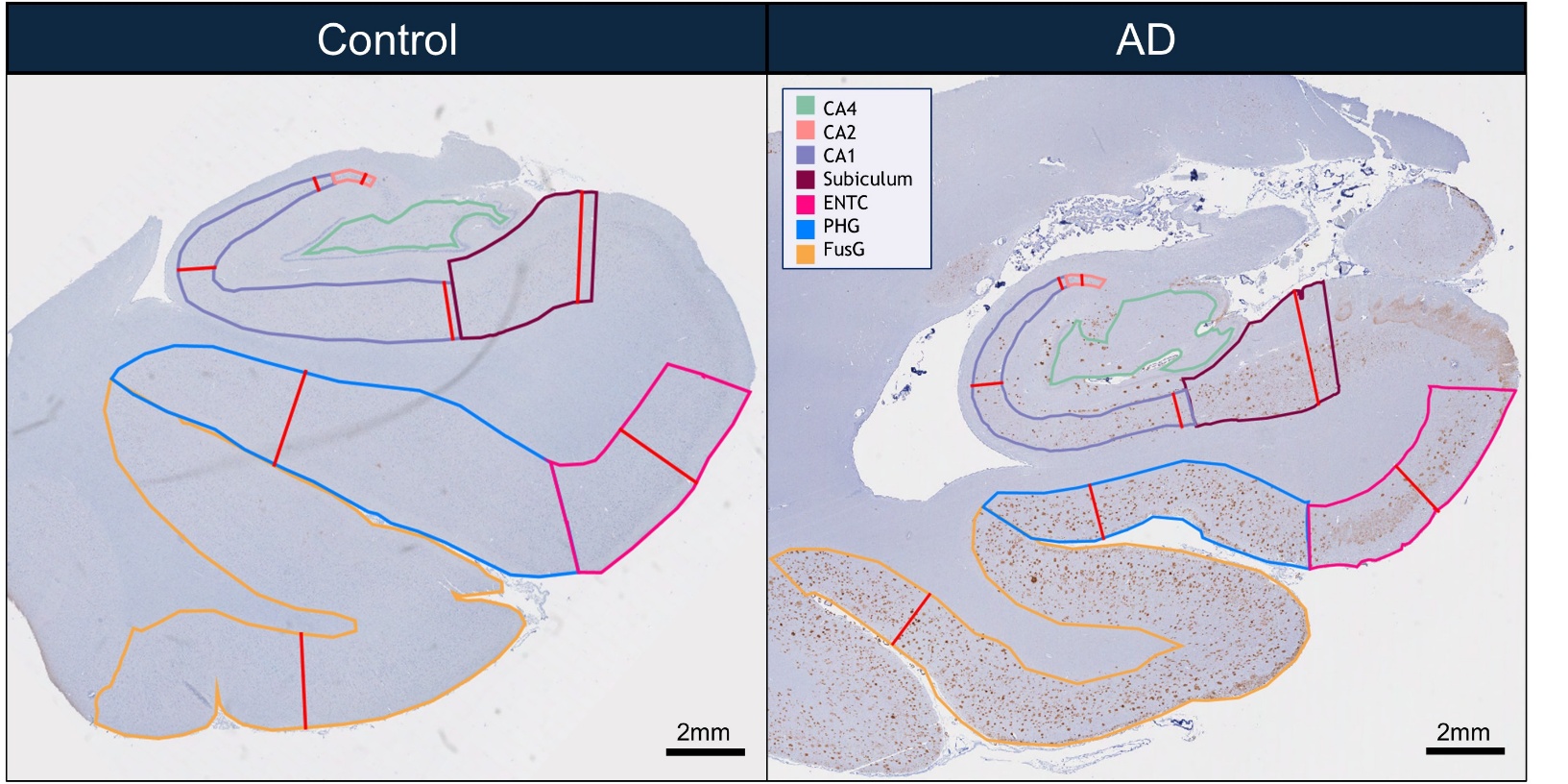
Supplementary Figures**

**Suppl. Fig. 1. Example of cortical thickness measurements in brightfield stained (amyloid-β) section in control and AD.** Cortical thickness was measured in all subfields (except CA4) in an amyloid-β stained tissue section adjacent to the section stained for synaptophysin, to correct for tissue atrophy in AD. The red lines drawn in the subfields represent the cortical thickness. As the thickness of the CA1 region varies a lot depending on the level (i.e. close to the CA2 or subiculum border), the average of 3 thickness measurements across the subfield were used. **Legend:** *AD: Alzheimer’s disease; CA: Cornu Ammonis; ENTC: entorhinal cortex; FusG: fusiform gyrus; PHG: parahippocampal gyrus.*

*
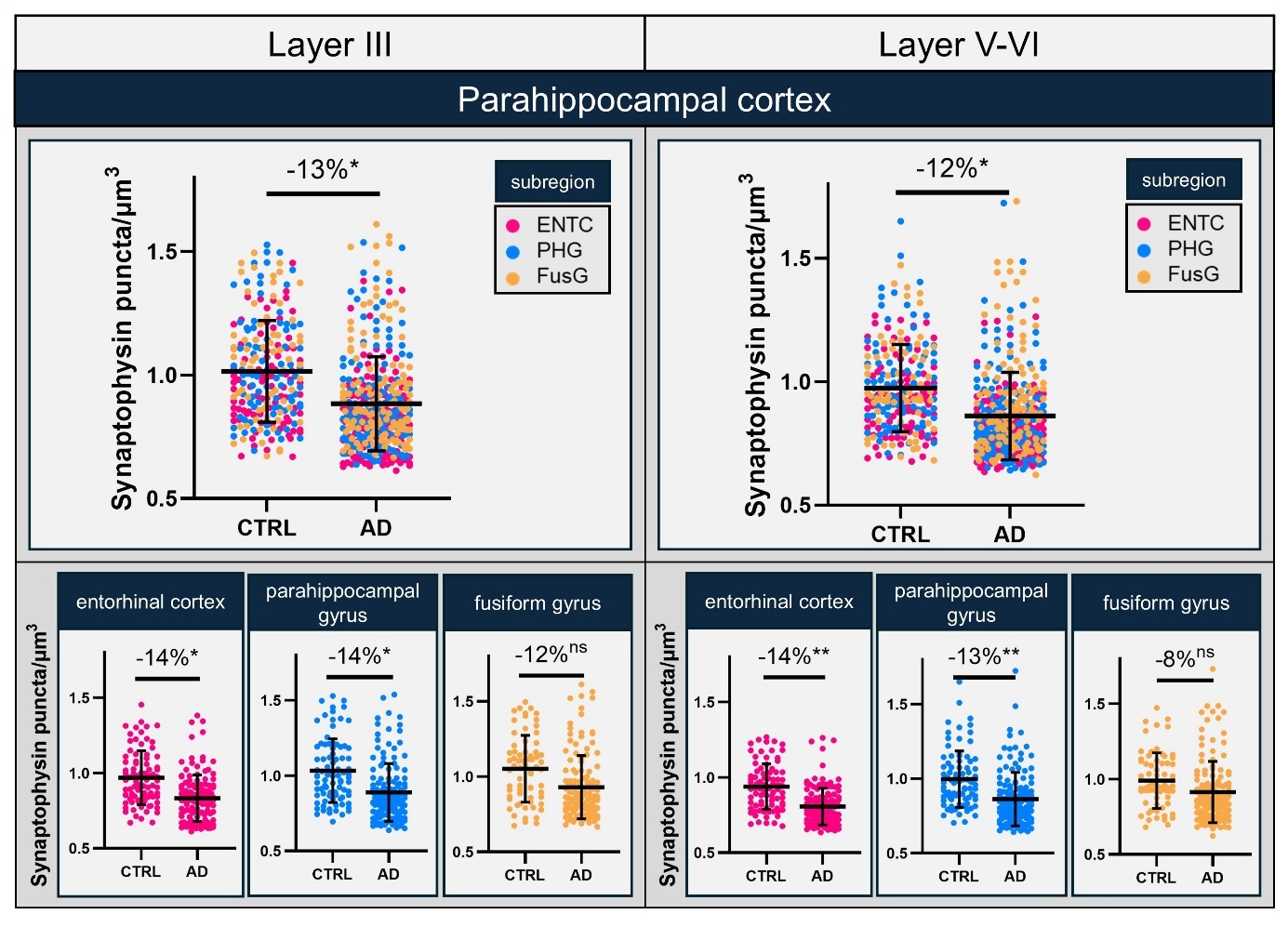
*

**Suppl. Fig. 2. Synaptic density differences between controls and AD in layer III and V-VI across parahippocampal subregions.** Each datapoint represents one measurement, and in each layer (superficial layer III and deep layer V-VI) 5 measurements were included per subregion for every case. The mean and standard deviations are depicted by the black lines in the graphs. All p-values are adjusted for multiple comparisons (7 subregions) with FDR-correction. ** p<0.001, *p<0.05. **Legend:** *AD: Alzheimer’s disease; ENTC: entorhinal cortex; FusG: fusiform gyrus; ns: not significant; PHG: parahippocampal gyrus.*

**
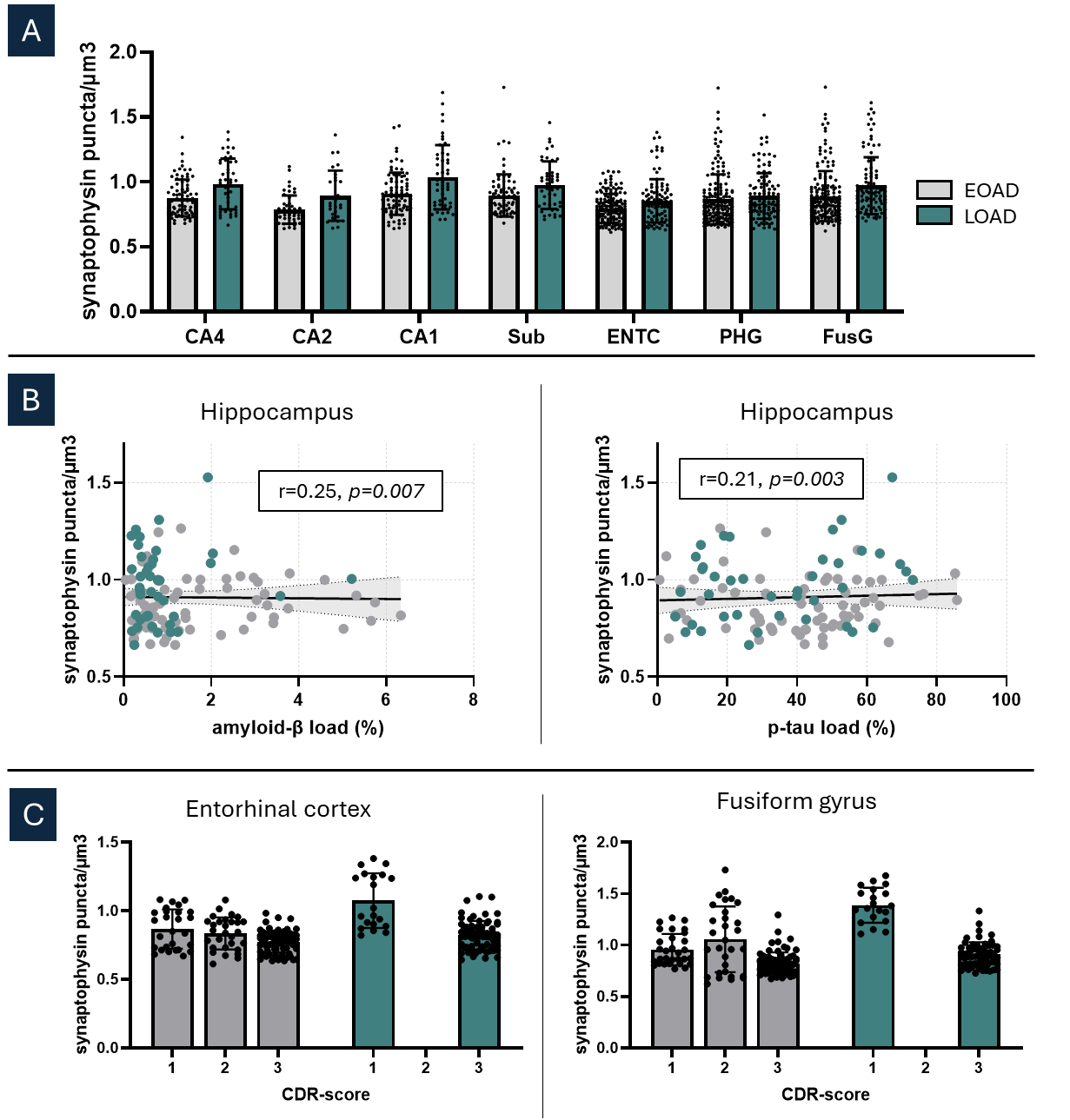
**
**Suppl. Fig. 3. Additional analyses in early vs. late-onset Alzheimer’s disease brain donors.** (A) Group differences in synaptophysin+ synaptic density across individual (para)hippocampal subregions between AD donors with early (EOAD) and late (LOAD) onset disease. Each datapoint represents one measurement, and multiple measurements per subregion were included for every case. The mean and standard deviation are depicted by the black lines in the graphs. No significant differences were observed between EOAD and LOAD. (B) Distribution of EOAD and LOAD donors in the significant correlations between synaptic density and amyloid-β and p-tau load in the hippocampal subregions grouped together, depicted in **Figure 4B**. The p-values are adjusted for multiple comparisons with FDR-correction. (C) Differences in synaptic density across dementia severity (CDR-score) in the entorhinal cortex and parahippocampal gyrus, the analysis suggested a trend towards lower synaptic density with increasing CDR scores in LOAD, but did not remain significant after FDR-correction for multiple comparisons. *Legend: CA: Cornu Ammonis; CDR: clinical dementia rating; ENTC: entorhinal cortex; EOAD: early-onset Alzheimer’s disease; FusG: fusiform gyrus; LOAD: late-onset Alzheimer’s disease; PHG: parahippocampal gyrus;* *Sub: subiculum.*

*
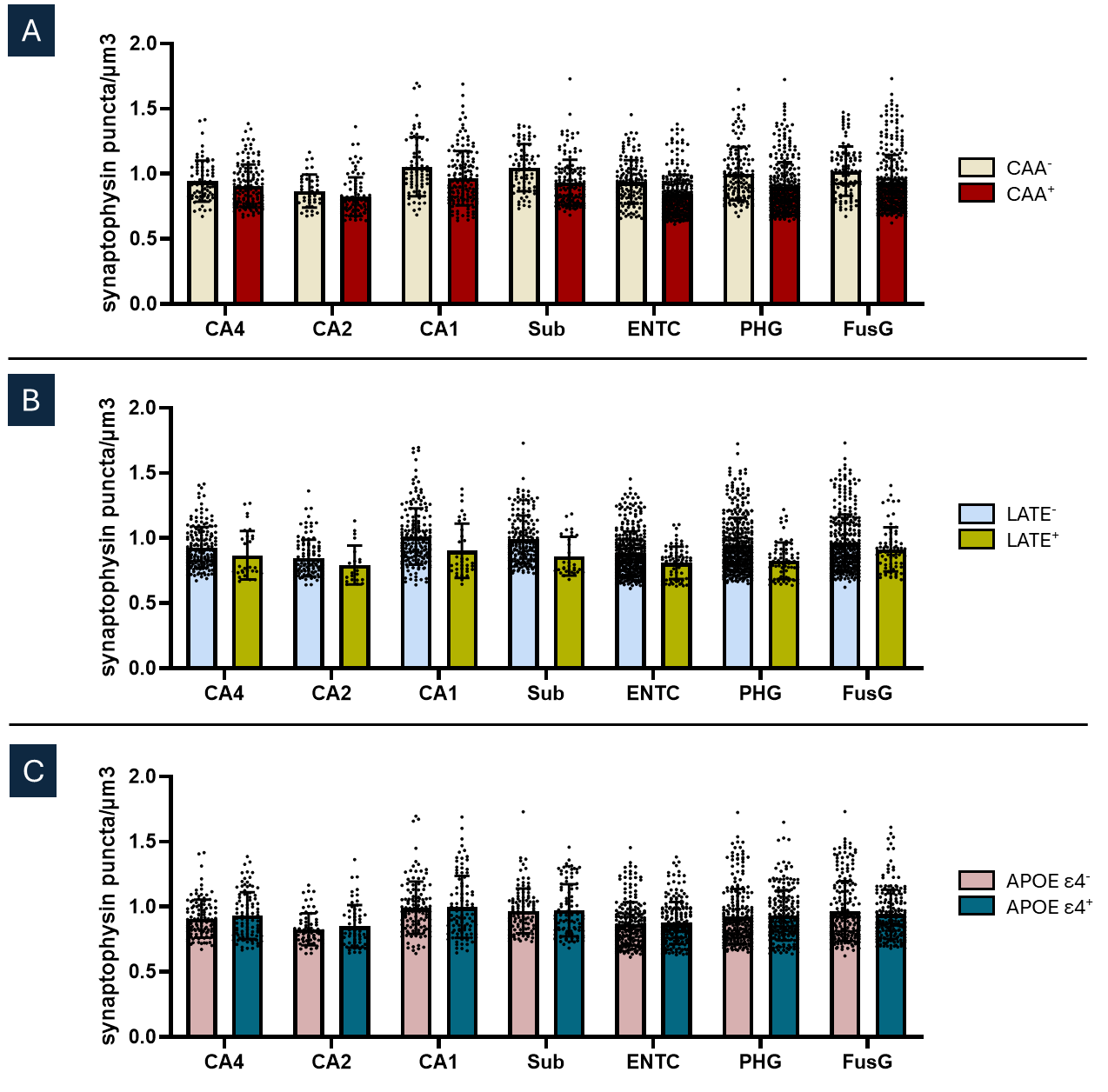
*

**Suppl. Fig. 4. Synaptic density differences between the presence or absence of CAA, LATE and APOE ε4.** Group differences in synaptophysin+ synaptic density across individual (para)hippocampal subregions between brain donors with (^+^) and without (^-^) CAA (A), LATE (B) an APOE ε4 allele (C). Each datapoint represents one measurement, and multiple measurements per subregion were included for every case. The mean and standard deviation are depicted by the black lines in the graphs. No p-values were significant after adjusting for multiple comparisons (7 subregions) with FDR-correction. *Legend: CA: Cornu Ammonis; CAA: cerebral amyloid angiopathy, ENTC: entorhinal cortex; FusG: fusiform gyrus; LATE: limbic-predominant age-related TDP-43 encephalopathy; PHG: parahippocampal gyrus;* *Sub: subiculum.*

**
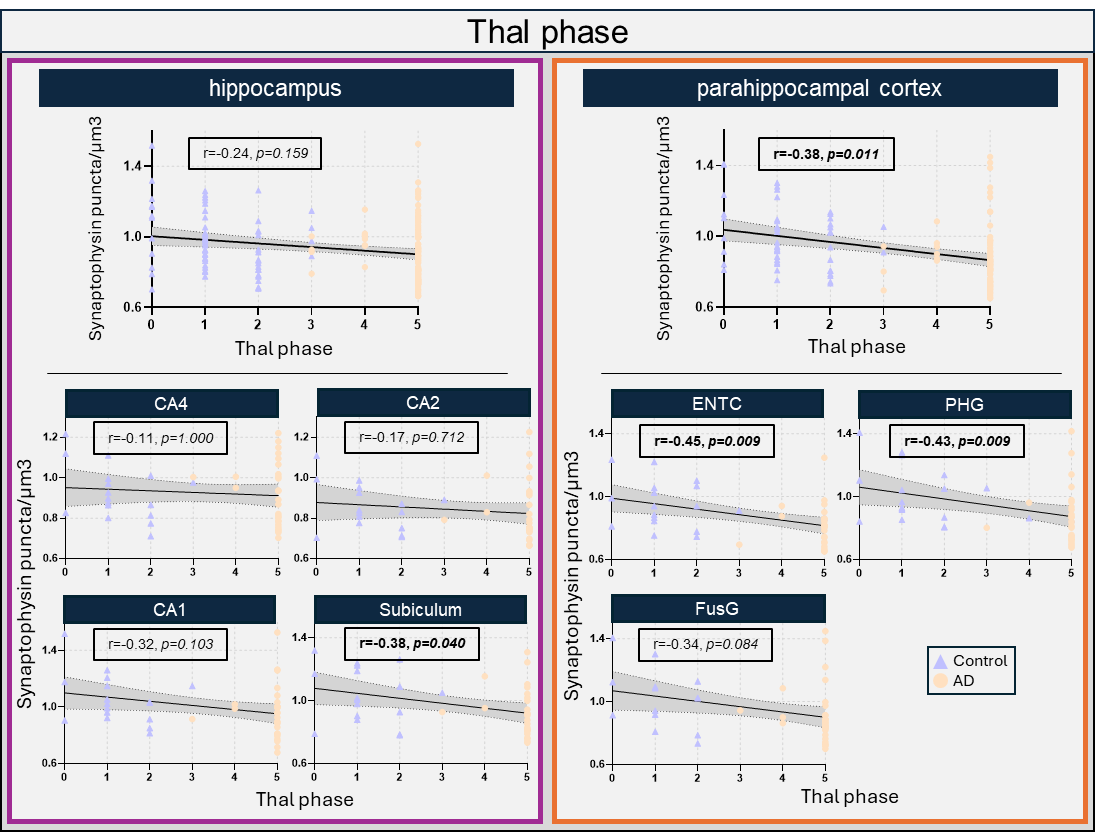
**

**Suppl. Fig. 5. Associations between Thal phase and synaptic density across the cohort.** Correlations between Thal phase and synaptic density in a combined cohort for hippocampal (purple box) and parahippocampal cortex (orange box) and individual subregions below. Each datapoint represents one averaged measurement per donor, color- and shape-coded for group. P-values were adjusted for multiple comparisons (7 subregions) with FDR-correction. Significant correlations were highlighted in bold. **Legend**: *AD: Alzheimer’s disease; CA: Cornu Ammonis; ENTC: entorhinal cortex; FusG: fusiform gyrus; PHG: parahippocampal gyrus; Sub: subiculum.*

**
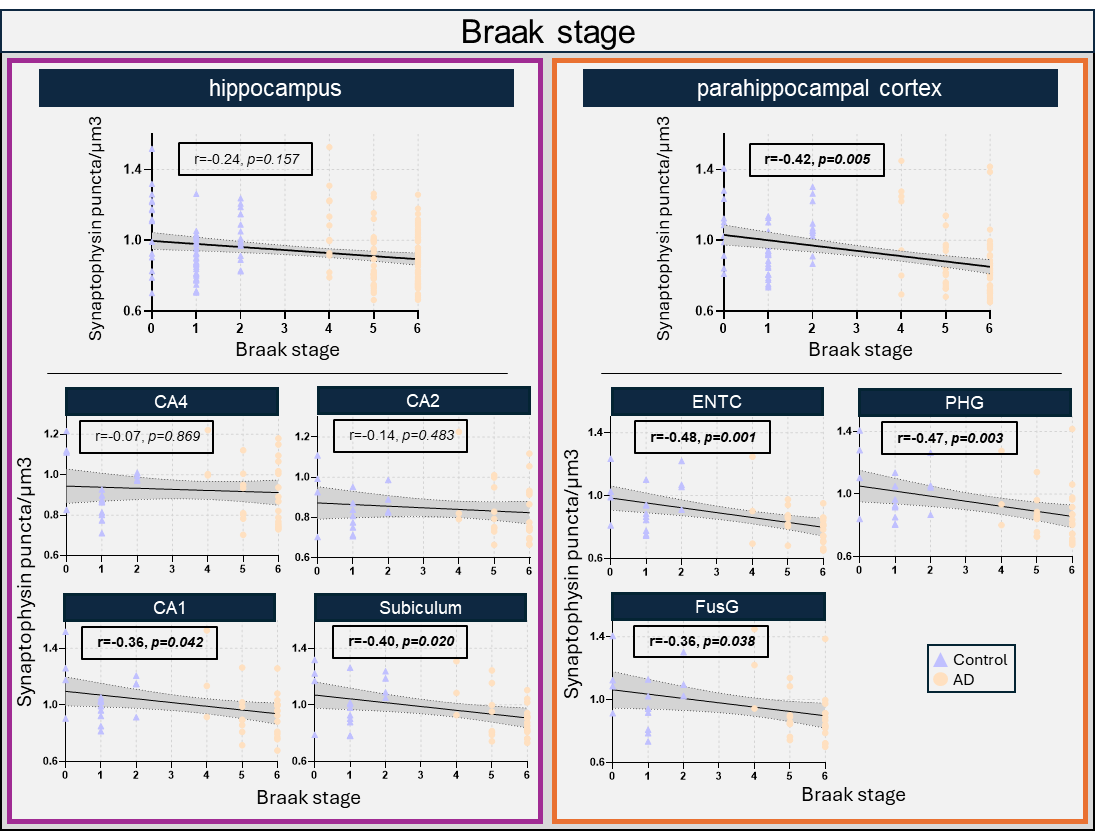
**

**Suppl. Fig. 6. Associations between Braak NFT stage and synaptic density across the cohort.** Correlations between Braak stage and synaptic density in a combined cohort for hippocampal (purple box) and parahippocampal cortex (orange box) and individual subregions below. Each datapoint represents one averaged measurement per donor, color- and shape-coded for group. P-values were adjusted for multiple comparisons (7 subregions) with FDR-correction. Significant correlations were highlighted in bold. **Legend**: *AD: Alzheimer’s disease; CA: Cornu Ammonis; ENTC: entorhinal cortex; FusG: fusiform gyrus; PHG: parahippocampal gyrus; Sub: subiculum.*

**
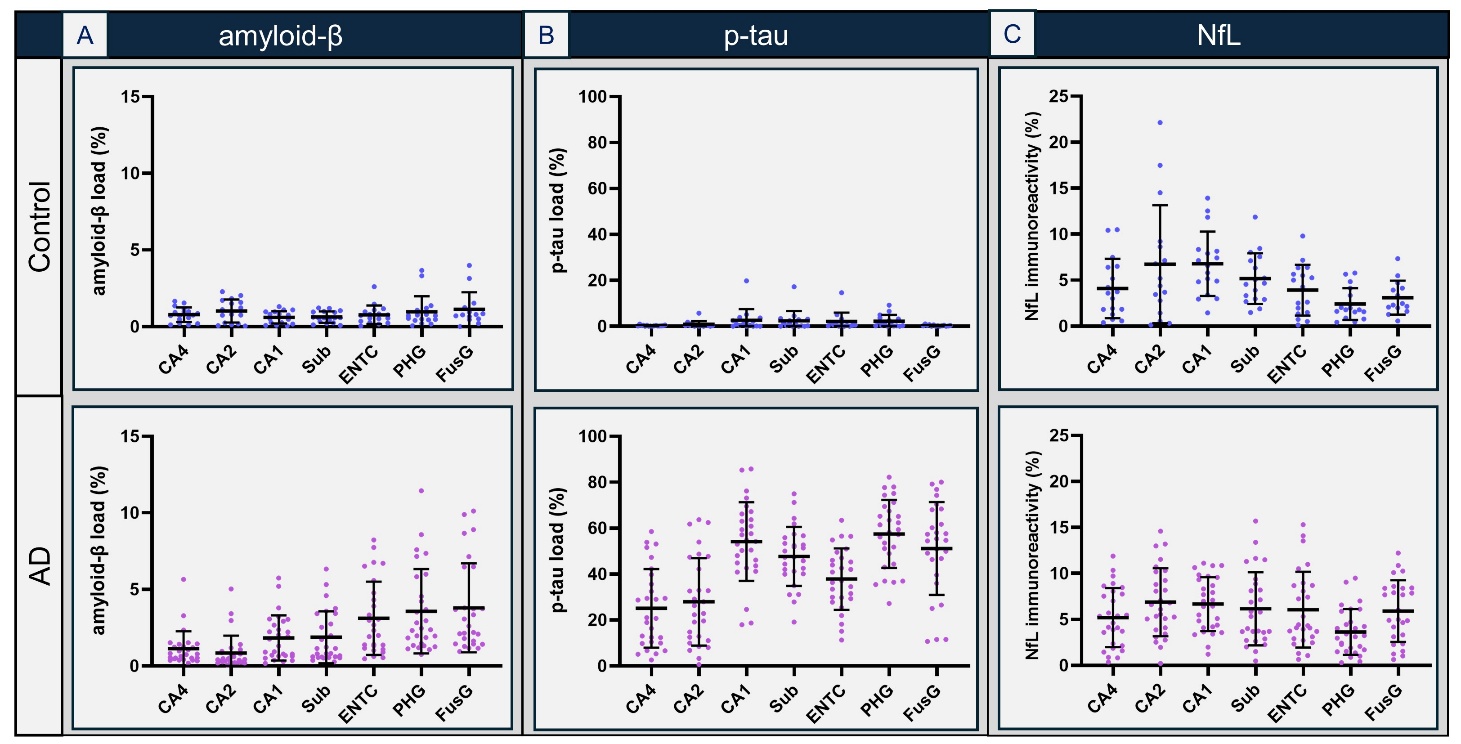
Suppl. Fig. 7. Distribution of neuropathological load and NfL immunoreactivity across (para)hippocampal subregions in controls and AD.** Distribution of amyloid-β **(A)** and p-tau load **(B)** and NfL immunoreactivity **(C)** across hippocampal and parahippocampal subregions in controls and AD. Each datapoint represents an average measurement per region per case. The mean and standard deviation are depicted by the black lines in the graphs. **Legend:** *AD: Alzheimer’s disease; CA: Cornu Ammonis; ENTC: entorhinal cortex; FusG: fusiform gyrus; NfL: neurofilament light chain; PHG: parahippocampal gyrus; Sub: subiculum.*

**
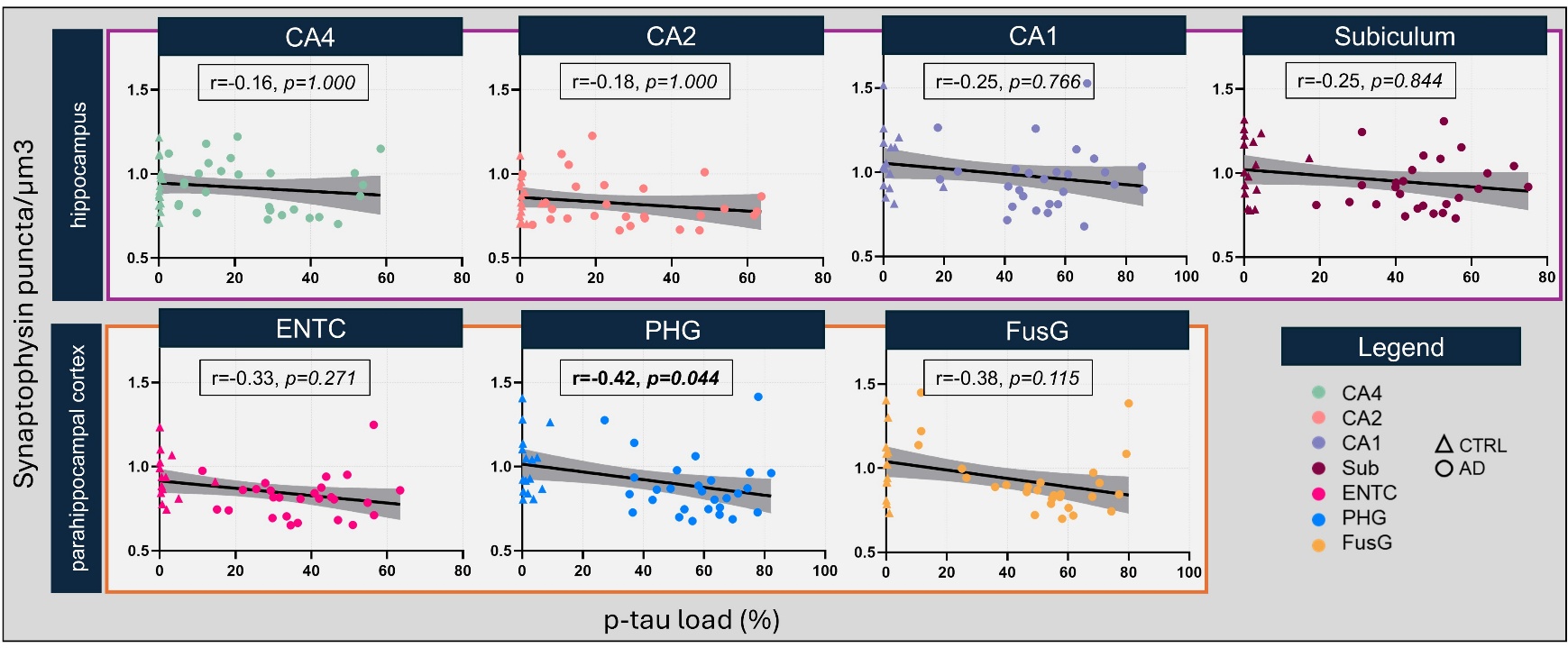
Suppl. Fig. 8. Subregional associations between p-tau load and synaptic density across the cohort.** Correlations between synaptic density and p-tau load are shown per subregion. Partial correlations p-values are adjusted for multiple comparisons (7 subregions) with FDR-correction. Synaptic density and p-tau load only significantly correlate in the parahippocampal gyrus. Each datapoint represents one averaged measurement, color-coded for subregion and shape-coded for group. ***Legend:*** *AD: Alzheimer’s disease; CA: Cornu Ammonis; CTRL: control; ENTC: entorhinal cortex; FusG: fusiform gyrus; NfL: neurofilament light chain; PHG: parahippocampal gyrus; Sub: subiculum*.

**
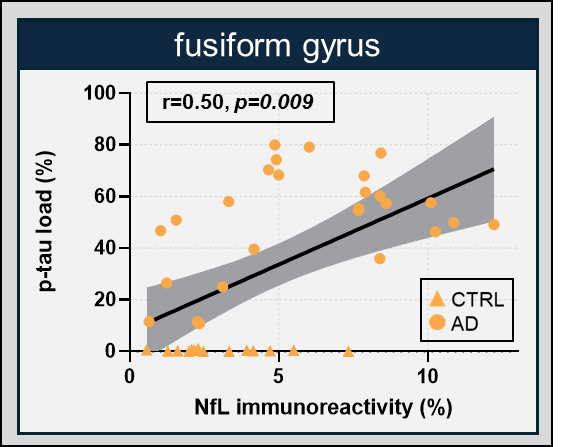
**

**Suppl. Fig. 9. Association between p-tau load and NfL immunoreactivity in the fusiform gyrus in the combined cohort.** Correlation between p-tau load and NfL immunoreactivity is shown for the fusiform gyrus. Partial correlation p-value is adjusted for multiple comparisons (7 subregions) with FDR-correction. Each datapoint represents one averaged measurement, shape-coded for group. **Legend:** *AD: Alzheimer’s disease; CTRL: control; NfL: neurofilament light chain.*
