# Supplementary material for: Distinct regional patterns of synaptic vulnerability across hippocampal and parahippocampal subregions in Alzheimer's disease
Source: Brain Pathol. 2026 Feb 9;36(4):e70081. doi: 10.1111/bpa.70081 (PMC13239152; doi:10.1111/bpa.70081)
Supplement: Supplementary file 2 — TABLE S1: Donor characteristics. TABLE S2: Information on primary antibodies. TABLE S3: Information on fluorescence Olympus VS200 scanning at 60× magnification. TABLE S4: Average thickness measurements of (para)hippocampal subregions and ratio used to normalize synaptic puncta in AD donors. TABLE S5: Characteristics of typical versus atypical AD. TABLE S6: Characteristics of early‐onset versus late‐onset AD. TABLE S7: Characteristics of APOE ε4 carrier (+) and non‐carriers (−) AD donors. TABLE S8: Detailed association model between synaptic density and amyloid‐β. p‐Values are FDR corrected for multiple subregions. Significant associations are in bold. TABLE S9: Detailed association model between synaptic density and p‐tau load. p‐Values are FDR corrected for multiple subregions. As none of the uncorrected p‐values were significant in the model of the controls, FDR‐correction was not possible. Significant associations are in bold. TABLE S10: Detailed association model between synaptic density and NfL immunoreactivity. p‐Values are FDR corrected for multiple subregions. Significant associations are in bold. TABLE S11: R‐values and p‐values before and after FDR‐correction of correlations between pathological markers in a combined cohort. As none of the uncorrected p‐values were significant for the NfL and amyloid‐β correlation, FDR‐correction was not possible. TABLE S12: Details of projection associations between amyloid‐β and synaptic density in interconnected parahippocampal and hippocampal subregions. As this analysis was exploratory, p‐values were not corrected for multiple comparisons. TABLE S13: Details of projection associations between p‐tau and synaptic density in interconnected parahippocampal and hippocampal subregions. As this analysis was exploratory, p‐values were not corrected for multiple comparisons. TABLE S14: Details of projection associations between NfL and synaptic density in interconnected parahippocampal and hippocampal subregions. As this analy [file BPA-36-e70081-s002.docx]

**Supplementary tables**

**Supplementary Table 1.** Donor characteristics.

| Donor ID # | Clinical diagnosis | Sex | Age at diagnosis (years) | Disease duration (years) | CDR | APOE genotype | Age at death (years) | PMD  (hr:min) | Cause of death | Thal phase^1^ | Braak NFT stage^2^ | Braak LB stage^3^ | ABC score^4^ | CAA type | LATE stage |
| --- | --- | --- | --- | --- | --- | --- | --- | --- | --- | --- | --- | --- | --- | --- | --- |
|  | CONTROLS | | | | | | | | | | | | | | |
| 1 | CTRL | M | - | - | - | 34 | 68 | 8:30 | Euthanasia | 2 | 1 | 0 | A1B1C0 | 1 | 0 |
| 2 | CTRL | F | - | - | - | 23 | 63 | 8:10 | Euthanasia | 0 | 0 | 0 | A0B0C0 | 0 | 0 |
| 3 | CTRL | M | - | - | - | 23 | 82 | 10:30 | Liver cirrhosis | 1 | 1 | 0 | A1B1C0 | 0 | 0 |
| 4 | CTRL | M | - | - | - | 33 | 85 | 9:22 | Euthanasia | 1 | 1 | 0 | A1B1C0 | 0 | 0 |
| 5 | CTRL | F | - | - | - | 33 | 76 | 7:50 | Euthanasia | 2 | 1 | 0 | A1B1C0 | 0 | 0 |
| 6 | CTRL | M | - | - | - | 23 | 67 | 8:10 | Liver cirrhosis | 1 | 1 | 0 | A1B1C0 | 1 | 0 |
| 7 | CTRL | F | - | - | - | 43 | 57 | 9:50 | Euthanasia | 1 | 0 | 0 | A1B0C0 | 0 | 0 |
| 8 | CTRL | F | - | - | - | 33 | 72 | 7:20 | Heart failure | 0 | 0 | 0 | A0B0C0 | 0 | 0 |
| 9 | CTRL | M | - | - | - | 34 | 59 | 8:00 | Euthanasia | 2 | 1 | 0 | A1B1C0 | 0 | 0 |
| 10 | CTRL | M | - | - | - | 23 | 77 | 11:25 | Pneumonia | 1 | 1 | 0 | A1B1C0 | 0 | 0 |
| 11 | CTRL | F | - | - | - | 33 | 78 | 10:00 | Unknown | 1 | 1 | 1 | A1B1C0 | 0 | 0 |
| 12 | CTRL | F | - | - | - | 33 | 59 | 8:10 | Euthanasia | 0 | 0 | 0 | A0B0C0 | 0 | 0 |
| 13 | CTRL | F | - | - | - | 34 | 71 | 6:50 | Lung carcinoma | 2 | 1 | 0 | A1B1C0 | 2 | 0 |
|  | AD |  |  |  |  |  |  |  |  |  |  |  |  |  |  |
| 14 | Amnestic | M | 60 | 2 | 2 | 33 | 60 | 8:21 | Euthanasia | 5 | 6 | 0 | A3B3C3 | 1 | 0 |
| 15 | Amnestic | M | 66 | 2 | 3 | 33 | 68 | 9:09 | Euthanasia | 5 | 5 | 0 | A3B3C3 | 1 | 0 |
| 16 | Amnestic | M | 62 | 7 | 3 | 34 | 69 | 11:33 | Pulmonary infection | 5 | 5 | 0 | A3B3C3 | 1 | 0 |
| 17 | Amnestic | M | 71 | 13 | 1 | 34 | 84 | 5:53 | Euthanasia | 4 | 5 | 0 | A3B2C2 | 0 | 0 |
| 18 | Amnestic | F | 79 | 1 | 1 | 33 | 80 | 7:03 | Epileptic seizure | 5 | 4 | 0 | A3B2C2 | 1 | 1 |
| 19 | Amnestic | M | 48 | 5 | NA | 33 | 53 | 9:00 | Dehydration | 5 | 6 | 0 | A3B3C3 | 2 | 0 |
| 20 | Amnestic | M | 54 | 10 | 3 | 34 | 64 | 7:55 | Euthanasia | 5 | 6 | 0 | A3B3C3 | 2 | 0 |
| 21 | Amnestic | M | 82 | 2 | NA | 33 | 84 | 6:16 | Medication overdose | 3 | 4 | 0 | A2B2C2 | 0 | 0 |
| 22 | Amnestic | M | 72 | 5 | NA | 44 | 77 | 6:17 | Euthanasia | 5 | 6 | 0 | A3B3C3 | 2 | 1 |
| 23 | Amnestic | M | 62 | 3 | NA | 34 | 65 | 9:18 | Myocardial infarction | 5 | 5 | 0 | A3B3C3 | 1 | 0 |
| 24 | Amnestic | M | 53 | 10 | 3 | 43 | 63 | 8:45 | Palliative sedation | 5 | 6 | 0 | A3B3C3 | 2 | 0 |
| 25 | Amnestic | F | 51 | 10 | NA | 44 | 61 | 7:40 | End-stage disease | 5 | 6 | 0 | A3B3C3 | 1 | 2 |
| 26 | Amnestic | F | 45 | 8 | 3 | 23 | 53 | 6:30 | Euthansia | 5 | 6 | 0 | A3B3C3 | 2 | 0 |
| 27 | Amnestic | M | 71 | 8 | NA | 33 | 79 | 8:05 | Unknown | 5 | 6 | 2 | A3B3C3 | 3 | 3 |
| 28 | B/D | F | 71 | 7 | 3 | 34 | 78 | 7:30 | Dehydration | 5 | 5 | 3 | A3B3C3 | 1 | 0 |
| 29 | PCA | M | 57 | 5 | 3 | 34 | 62 | 8:09 | Palliative sedation | 5 | 6 | 0 | A3B3C3 | 1 | 0 |
| 30 | B/D | M | 35 | 2 | 1 | 23 | 37 | 11:07 | Euthanasia | 5 | 6 | 0 | A3B3C3 | 3 | 0 |
| 31 | B/D | M | 56 | 2 | NA | 43 | 58 | 8:55 | Cachexia | 5 | 6 | 0 | A3B3C3 | 2 | 0 |
| 32 | PCA | M | 60 | 7 | 3 | 34 | 67 | 6:21 | Cachexia | 5 | 6 | 0 | A3B3C3 | 1 | 0 |
| 33 | B/D | M | 74 | 3 | 1 | 34 | 77 | 7:00 | Euthanasia | 5 | 4 | 0 | A3B2C2 | 1 | 0 |
| 34 | B/D | F | 57 | 2 | 3 | 34 | 59 | 3:34 | Dysphagia | 5 | 5 | - | A3B3C3 | 2 | 0 |
| 35 | lvPPA | F | 67 | 5 | 3 | 23 | 72 | 5:05 | End-stage disease | 5 | 6 | 0 | A3B3C3 | 1 | 0 |
| 36 | B/D | F | 72 | 1 | 3 | 34 | 73 | 6:15 | Pneumothorax | 5 | 6 | 0 | A3B3C3 | 1 | 1 |
| 37 | PCA | F | 58 | 2 | NA | 33 | 60 | 10:50 | Euthanasia | 5 | 6 | 0 | A3B3C3 | 1 | 0 |
| 38 | PCA | M | 65 | 3 | 3 | 43 | 68 | 6:22 | Dehydration | 5 | 6 | 0 | A3B3C3 | 1 | 0 |
| 39 | lvPPA | M | 70 | 5 | 3 | 44 | 75 | 8:35 | Epileptic seizure | 5 | 6 | 0 | A3B3C3 | 3 | 3 |
| 40 | lvPPA | F | 66 | 1 | 3 | 33 | 67 | 8:15 | End-stage disease | 5 | 5 | 0 | A3B3C3 | 1 | 0 |
| 41 | NA | M | 58 | 12 | NA | 44 | 70 | 5:48 | End-stage disease | 5 | 5 | 0 | A3B3C3 | 1 | 2 |
| 42 | NA | M | 58 | 7 | 2 | 33 | 65 | 7:48 | Cardiac arrest | 4 | 5 | 0 | A3B3C3 | 0 | 0 |

**Legend:** *AD: Alzheimer’s disease; B/D: behavioral/dysexecutive; CAA: cerebral amyloid angiopathy; CDR: clinical dementia rating; CTRL: control; F: female; HP: hippocampus; ICV: intracranial volume; LATE: limbic-predominant age-related TDP-43 encephalopathy; LB: Lewy bodies; lvPPA; logopenic variant primary progressive aphasia; M: male; MTA: medial temporal lobe atrophy; NA: not applicable; NFT: neurofibrillary tangles; PCA: posterior cortical atrophy; PD: Parkinson’s disease; PDD: Parkinson’s disease dementia; PMD: post-mortem delay.*

**Supplementary Table 2.** Information on primary antibodies.

| Primary antibody | Antigen | Species | Company | Dilution | Incubation | Antigen retrieval | Detection method |
| --- | --- | --- | --- | --- | --- | --- | --- |
| Synaptophysin, clone DAK-SYNAP | Recombinant protein of C-terminal cytoplasmic domain | Mouse igG1 | Agilent DAKO, Santa Clara, USA | 1:50 | 4°C o.n. | Tris EDTA buffer (pH 9.0) in steam cooker | FL  DoAM Alexa488 |
| Aβ, clone 4G8 | Aβ amino acid sequence 17-24 | Mouse igG2b | BioLegend, San Diego, USA | 1:8000 | 4°C o.n. | Citrate buffer (pH 6.0) in steam cooker | BF  EnVision (HRP) |
| p-tau, clone AT8 | Tau phosphorylated at Ser202 and Thr205 | Mouse igG1 | ThermoFisher, Pittsburgh, USA | 1:800 | 4°C o.n. | Citrate buffer (pH 6.0) in steam cooker | BF  EnVision (HRP) |
| NfL | Immunogen corresponds to AA 1 to 284 (with AA 200-292 missing) | Rabbit igG | Synaptic systems, Göttingen, Germany | 1:600 | 4°C o.n. | Tris EDTA buffer (pH 9.0) in steam cooker | BF  EnVision (HRP) |
| Secondary antibody | **Host species** | **Target species** | **Company** | **Dilution** | **Incubation** | **Antigen retrieval** | **Detection method** |
| DoAM Alexa488 | Donkey IgG | Mouse | ThermoFisher, Pittsburgh, USA | 1:200 | 2 hrs at RT | / | Fluorochrome |

**Legend**: *BF: brightfield; DoAM: donkey anti-mouse; FL: fluorescence; hrs: hours; NfL: neurofilament light chain; o.n.: overnight; RT: room temperature.*

**Supplementary Table 3.** Information on fluorescence Olympus VS200 scanning at 60x magnification.

| Channel | Target | Excitation wavelength | Filter | Exposure time |
| --- | --- | --- | --- | --- |
| DAPI | Nuclei | 378/52 nm | 432/36 nm | 1.5 ms |
| Alexa 488 | Synaptophysin | 474/27 nm | 515/36 nm | 50 ms |
| Autofluorescence | Autofluorescence | 474/27 nm | 595/31 nm | 50 ms |

**Supplementary Table 4.** Average thickness measurements of (para)hippocampal subregions and ratio used to normalize synaptic puncta in AD donors.

| Subregion | Control | AD | Ratio |
| --- | --- | --- | --- |
| CA2 | 297 µm | 284 µm | 0.956 |
| CA1 | 787µm | 776 µm | 0.986 |
| Subiculum | 2748 µm | 2691 µm | 0.979 |
| ENTC | 2383 µm | 2249 µm | 0.944 |
| PHG | 2655 µm | 2471 µm | 0.931 |
| FusG | 2404 µm | 2312 µm | 0.962 |

**Supplementary Table 5.** Characteristics of typical vs. atypical AD

|  | Typical AD | Atypical AD | P-value |
| --- | --- | --- | --- |
| N | 13 | 14 |  |
| Sex F/M (% F) | 3/10 (23%) | 6/8 (43%) | 0.420 |
| Age at diagnosis  years, mean [range] | 58 [45-73] | 62 [32-73] | 0.300 |
| Disease duration  years, mean [range] | 9 [2-23] | 5 [1-13] | **0.018** |
| Age at death  years, mean [range] | 67 [53-84] | 67 [37-84] | 0.983 |
| CDR  median (N) [range] | 2.5 (N=8)  [1-3] | 3 (N=13)  [1-3] | 0.684 |
| APOE genotype N (%)  ε4 non-carrier  ε4 heterozygous  ε4 homozygous | 7 (54%)  4 (31%)  2 (15%) | 5 (36%)  8 (57%)  1 (7%) | 0.466 |
| Pathological characteristics | | | |
| Thal phase N  0/1/2/3/4/5 | 0/0/0/1/1/11 | 0/0/0/0/1/13 | 0.730 |
| Braak NFT stage N  0/1/2/3/4/5/6 | 0/0/0/0/2/4/7 | 0/0/0/0/1/4/9 | 0.865 |
| Braak α-synuclein stage N  0/1/2/3/4/5/6 | 12/1/0/0/0/0/0 | 13/0/0/1/0/0/0 | 1.000 |
| CAA N (%)  Absent/type 1/type 2 | 12 (92%)  1/9/3 | 14 (100%)  0/9/5 | 0.678 |
| LATE N (%)  Stage 0/1/2/3 | 4 (31%)  9/2/1/1 | 2 (14%)  12/1/0/1 | 0.385 |

**Legend: *AD:*** *Alzheimer’s disease; CAA: cerebral amyloid angiopathy; CDR: clinical dementia rating; F: female; LATE: limbic-predominant age-related TDP-43 encephalopathy; M: male; NFT: neurofibrillary tangles.*

**Supplementary Table 6.** Characteristics of early-onset vs. late-onset AD

|  | EOAD | LOAD | P-value |
| --- | --- | --- | --- |
| N | 17 | 11 |  |
| Sex F/M (% F) | 4/13 (31%) | 5/6 (45%) | 0.409 |
| Age at diagnosis  years, mean [range] | 54 [32-62] | 70 [65-73] | **<0.001** |
| Disease duration  years, mean [range] | 8 [2-23] | 6 [1-13] | 0.313 |
| Age at death  years, mean [range] | 62 [37-84] | 75 [67-84] | **<0.001** |
| CDR  median (N) [range] | 2.5 (N=12)  [1-3] | 3 (N=9)  [1-3] | 0.314 |
| APOE genotype N (%)  ε4 non-carrier  ε4 heterozygous  ε4 homozygous | 8 (47%)  7 (41%)  2 (12%) | 4 (36%)  5 (45%)  2 (18%) | 0.819 |
| Pathological characteristics | | | |
| Thal phase N  0/1/2/3/4/5 | 0/0/0/1/2/14 | 0/0/0/1/2/11 | 0.505 |
| Braak NFT stage N  0/1/2/3/4/5/6 | 0/0/0/0/1/7/9 | 0/0/0/0/2/2/7 | 0.412 |
| Braak α-synuclein stage N  0/1/2/3/4/5/6 | 17/0/0/0/0/0/0 | 9/1/0/1/0/0/0 | 0.146 |
| CAA N (%)  Absent/type 1/type 2 | 16 (94%)  1/12/4 | 11 (100%)  0/7/4 | 0.803 |
| LATE N (%)  Stage 0/1/2/3 | 2 (12%)  15/0/2/0 | 5 (45%)  6/3/0/2 | 0.076 |

**Legend: *AD:*** *Alzheimer’s disease; CAA: cerebral amyloid angiopathy; CDR: clinical dementia rating; EOAD: early-onset Alzheimer’s disease; F: female; LATE: limbic-predominant age-related TDP-43 encephalopathy; LOAD: late-onset Alzheimer’s disease; M: male; NFT: neurofibrillary tangles.*

**Supplementary Table 7.** Characteristics of APOE ε4 carrier (+) and non-carriers (–) AD donors

|  | APOE ε4 + | APOE ε4 – | P-value |
| --- | --- | --- | --- |
| N | 16 | 12 |  |
| Sex F/M (% F) | 4/12 (25%) | 5/7 (42%) | 0.432 |
| Age at diagnosis  years, mean [range] | 62 [51-73] | 58 [32-73] | 0.323 |
| Disease duration  years, mean [range] | 7 [1-13] | 7 [1-23] | 0.738 |
| Age at death  years, mean [range] | 69 [58-84] | 65 [37-84] | 0.292 |
| CDR  median (N) [range] | 3 (N=12)  [1-3] | 2 (N=9)  [1-3] | 0.161 |
| APOE genotype N (%)  ε4 non-carrier  ε4 heterozygous  ε4 homozygous | 0 (0%)  12 (75%)  4 (25%) | 12 (100%)  0 (0%)  0 (0%) | **<0.001** |
| Pathological characteristics | | | |
| Thal phase N  0/1/2/3/4/5 | 0/0/0/0/1/15 | 0/0/0/1/1/10 | 0.707 |
| Braak NFT stage N  0/1/2/3/4/5/6 | 0/0/0/0/1/6/9 | 0/0/0/0/2/3/7 | 0.552 |
| Braak α-synuclein stage N  0/1/2/3/4/5/6 | 15/0/0/1/0/0/0 | 11/1/0/0/0/0/0 | 0.683 |
| CAA N (%)  Absent/type 1/type 2 | 16 (100%)  0/12/4 | 11 (92%)  1/7/4 | 0.517 |
| LATE N (%)  Stage 0/1/2/3 | 5 (31%)  11/2/2/1 | 2 (17%)  10/1/0/1 | 0.662 |

**Legend: *AD:*** *Alzheimer’s disease; CAA: cerebral amyloid angiopathy; CDR: clinical dementia rating; F: female; LATE: limbic-predominant age-related TDP-43 encephalopathy; M: male; NFT: neurofibrillary tangles.*

**Supplementary Table 8.** **Detailed association model between synaptic density and amyloid-β.** P-values are FDR corrected for multiple subregions. Significant associations are in bold.

| **Group** | **Subregion** | **r-value** | **R^2^** | **95% CI** | **p-value** |
| --- | --- | --- | --- | --- | --- |
| Controls | **Hippocampus** | **-0.52** | **27.3%** | **[-0.108, 0.039]** | **<0.001** |
|  | Parahippocampal cortex | 0.10 | 1.0% | [-0.013, 0.029] | 0.906 |
| AD | **Hippocampus** | **0.25** | **6.3%** | **[0.005, 0.022]** | **0.003** |
|  | Parahippocampal cortex | 0.22 | 4.9% | [-0.002, 0.015] | 0.284 |

**Legend:** *AD: Alzheimer’s disease; CI: confidence interval; R^2^: determination coefficient*

**Supplementary Table 9. Detailed association model between synaptic density and p-tau load.** P-values are FDR corrected for multiple subregions. As none of the uncorrected p-values were significant in the model of the controls, FDR-correction was not possible. Significant associations are in bold.

| **Group** | **Subregion** | **r-value** | **R^2^** | **95% CI** | **p-value** |
| --- | --- | --- | --- | --- | --- |
| Controls | Hippocampus | 0.15 | 2.1% | [-0.002, 0.008] | - |
|  | Parahippocampal cortex | -0.17 | 1.5% | [-0.009, 0.000] | - |
| AD | **Hippocampus** | **0.21** | **4.5%** | **[0.000, 0.001]** | **0.003** |
|  | Parahippocampal cortex | 0.12 | 3.0% | [0.000, 0.001] | 0.394 |

**Legend:** *AD: Alzheimer’s disease; CI: confidence interval; R^2^: determination coefficient*

**Supplementary Table 10. Detailed association model between synaptic density and NfL immunoreactivity.** P-values are FDR corrected for multiple subregions. Significant associations are in bold.

| **Group** | **Subregion** | **r-value** | **R^2^** | **95% CI** | **p-value** |
| --- | --- | --- | --- | --- | --- |
| Controls + AD | CA4 | -0.11 | 1.1% | [-0.009, 0.004] | 1.000 |
|  | CA2 | -0.13 | 1.8% | [-0.006, 0.003] | 1.000 |
|  | CA1 | 0.00 | 0.0% | [-0.008, 0.008] | 1.000 |
|  | Subiculum | -0.08 | 0.6% | [-0.008, 0.005] | 1.000 |
|  | Entorhinal cortex | -0.03 | 0.1% | [-0.007, 0.006] | 1.000 |
|  | Parahippocampal gyrus | -0.15 | 2.3% | [-0.018, 0.006] | 1.000 |
|  | **Fusiform gyrus** | **-0.44** | 19.0% | [-0.020, -0.004] | **0.044** |

**Legend:** *AD: Alzheimer’s disease; CA: Cornu Ammonis; CI: confidence interval; R^2^: determination coefficient*

**Supplementary Table 11.** R-values and p-values before and after FDR-correction of correlations between pathological markers in a combined cohort. As none of the uncorrected p-values were significant for the NfL and amyloid-β correlation, FDR-correction was not possible.

|  | **Subregion** | **r-value** | **uncorrected p-value** | **FDR-corrected p-value** |
| --- | --- | --- | --- | --- |
| **NfL - p-tau** | CA4 | 0.11 | 0.480 | 1 |
|  | CA2 | 0.04 | 0.805 | 1 |
|  | CA1 | 0.01 | 0.955 | 1 |
|  | Subiculum | 0.16 | 0.313 | 1 |
|  | Entorhinal cortex | 0.03 | 0.856 | 1 |
|  | Parahippocampal gyrus | 0.27 | 0.089 | 0.625 |
|  | **Fusiform gyrus** | **0.50** | **0.001** | **0.009** |
| **NfL - amyloid-β** | CA4 | 0.08 | 0.603 | - |
|  | CA2 | 0.10 | 0.528 | - |
|  | CA1 | -0.08 | 0.604 | - |
|  | Subiculum | 0.11 | 0.489 | - |
|  | Entorhinal cortex | -0.17 | 0.275 | - |
|  | Parahippocampal gyrus | 0.08 | 0.629 | - |
|  | Fusiform gyrus | 0.11 | 0.524 | - |
| **p-tau - amyloid-β** | CA4 | 0.08 | 0.050 | 0.070 |
|  | CA2 | 0.10 | 0.586 | 0.820 |
|  | **CA1** | **-0.08** | **0.009** | **0.012** |
|  | **Subiculum** | **0.11** | **0.001** | **0.001** |
|  | **Entorhinal cortex** | **-0.17** | **<0.001** | **0.001** |
|  | **Parahippocampal gyrus** | **0.08** | **0.001** | **0.001** |
|  | **Fusiform gyrus** | **0.11** | **<0.001** | **<0.001** |

**Supplementary Table 12. Details of projection associations between amyloid-β and synaptic density in interconnected parahippocampal and hippocampal subregions.** As this analysis was exploratory, p-values were not corrected for multiple comparisons.

| **Group** | **Projecting region (amyloid-β)** | **Target region (synaptic density)** | **r-value** | **p-value** |
| --- | --- | --- | --- | --- |
| Controls | Fusiform gyrus | Parahippocampal gyrus | -0.11 | 0.793 |
|  | Parahippocampal gyrus | Entorhinal cortex | 0.31 | 0.463 |
|  | Entorhinal cortex | CA1 | -0.43 | 0.292 |
|  | Entorhinal cortex | Subiculum | 0.36 | 0.383 |
|  | Subiculum | CA1 | -0.06 | 0.889 |
|  | CA2 | CA1 | 0.05 | 0.908 |
|  | CA1 | CA2 | 0.37 | 0.372 |
|  | CA1 | Subiculum | 0.41 | 0.318 |
|  | Subiculum | Entorhinal cortex | 0.42 | 0.295 |
|  | Entorhinal cortex | Parahippocampal gyrus | -0.17 | 0.691 |
|  | Parahippocampal gyrus | Fusiform gyrus | 0.24 | 0.571 |
| AD | Fusiform gyrus | Parahippocampal gyrus | 0.14 | 0.537 |
|  | Parahippocampal gyrus | Entorhinal cortex | -0.09 | 0.700 |
|  | Entorhinal cortex | CA1 | 0.03 | 0.910 |
|  | Entorhinal cortex | Subiculum | 0.02 | 0.915 |
|  | Subiculum | CA1 | -0.07 | 0.751 |
|  | CA2 | CA1 | -0.04 | 0.851 |
|  | CA1 | CA2 | 0.22 | 0.305 |
|  | CA1 | Subiculum | 0.14 | 0.525 |
|  | Subiculum | Entorhinal cortex | 0.01 | 0.954 |
|  | Entorhinal cortex | Parahippocampal gyrus | 0.17 | 0.439 |
|  | Parahippocampal gyrus | Fusiform gyrus | 0.05 | 0.820 |

**Supplementary Table 13. Details of projection associations between p-tau and synaptic density in interconnected parahippocampal and hippocampal subregions.** As this analysis was exploratory, p-values were not corrected for multiple comparisons.

| **Group** | **Projecting region (p-tau)** | **Target region (synaptic density)** | **r-value** | **p-value** |
| --- | --- | --- | --- | --- |
| Controls | Fusiform gyrus | Parahippocampal gyrus | -0.64 | 0.086 |
|  | Parahippocampal gyrus | Entorhinal cortex | 0.49 | 0.223 |
|  | Entorhinal cortex | CA1 | -0.27 | 0.516 |
|  | Entorhinal cortex | Subiculum | -0.19 | 0.645 |
|  | Subiculum | CA1 | 0.04 | 0.929 |
|  | CA2 | CA1 | -0.09 | 0.830 |
|  | CA1 | CA2 | 0.11 | 0.790 |
|  | CA1 | Subiculum | 0.68 | 0.062 |
|  | **Subiculum** | **Entorhinal cortex** | **0.78** | **0.023** |
|  | Entorhinal cortex | Parahippocampal gyrus | -0.15 | 0.716 |
|  | Parahippocampal gyrus | Fusiform gyrus | 0.19 | 0.646 |
| AD | Fusiform gyrus | Parahippocampal gyrus | -0.12 | 0.600 |
|  | Parahippocampal gyrus | Entorhinal cortex | -0.22 | 0.306 |
|  | Entorhinal cortex | CA1 | 0.11 | 0.617 |
|  | Entorhinal cortex | Subiculum | 0.04 | 0.863 |
|  | Subiculum | CA1 | 0.25 | 0.258 |
|  | CA2 | CA1 | -0.23 | 0.283 |
|  | CA1 | CA2 | -0.01 | 0.972 |
|  | CA1 | Subiculum | 0.13 | 0.542 |
|  | Subiculum | Entorhinal cortex | 0.26 | 0.233 |
|  | Entorhinal cortex | Parahippocampal gyrus | 0.17 | 0.426 |
|  | Parahippocampal gyrus | Fusiform gyrus | -0.14 | 0.513 |

**Supplementary Table 14. Details of projection associations between NfL and synaptic density in interconnected parahippocampal and hippocampal subregions.** As this analysis was exploratory, p-values were not corrected for multiple comparisons.

| **Group** | **Projecting region (NfL)** | **Target region (synaptic density)** | **r-value** | **p-value** |
| --- | --- | --- | --- | --- |
| Controls + AD | **Fusiform gyrus** | **Parahippocampal gyrus** | **-0.35** | **0.039** |
|  | Parahippocampal gyrus | Entorhinal cortex | -0.19 | 0.281 |
|  | Entorhinal cortex | CA1 | -0.09 | 0.584 |
|  | Entorhinal cortex | Subiculum | -0.09 | 0.590 |
|  | Subiculum | CA1 | 0.00 | 0.990 |
|  | CA2 | CA1 | -0.03 | 0.877 |
|  | CA1 | CA2 | 0.05 | 0.789 |
|  | CA1 | Subiculum | -0.19 | 0.276 |
|  | Subiculum | Entorhinal cortex | -0.03 | 0.846 |
|  | Entorhinal cortex | Parahippocampal gyrus | -0.13 | 0.464 |
|  | **Parahippocampal gyrus** | **Fusiform gyrus** | **-0.34** | **0.045** |

**Supplementary Table 15. Details of projection associations between amyloid-β in the superficial layers of the entorhinal cortex and synaptic density in interconnected hippocampal subregions.** As this analysis was exploratory, p-values were not corrected for multiple comparisons.

| **Group** | **Projecting region (amyloid-β)** | **Target region (synaptic density)** | **r-value** | **p-value** |
| --- | --- | --- | --- | --- |
| Controls | **Superficial layers of entorhinal cortex** | **CA1** | **-0.543** | **0.045** |
|  | Superficial layers of entorhinal cortex | Subiculum | 0.01 | 0.966 |
| AD | Superficial layers of entorhinal cortex | CA1 | 0.04 | 0.846 |
|  | Superficial layers of entorhinal cortex | Subiculum | 0.04 | 0.837 |

**Supplementary Table 16. Details of projection associations between p-tau in the superficial layers of the entorhinal cortex and synaptic density in interconnected hippocampal subregions.** As this analysis was exploratory, p-values were not corrected for multiple comparisons.

| **Group** | **Projecting region (p-tau)** | **Target region (synaptic density)** | **r-value** | **p-value** |
| --- | --- | --- | --- | --- |
| Controls | Superficial layers of entorhinal cortex | CA1 | 0.37 | 0.192 |
|  | Superficial layers of entorhinal cortex | Subiculum | 0.41 | 0.151 |
| AD | Superficial layers of entorhinal cortex | CA1 | 0.06 | 0.796 |
|  | Superficial layers of entorhinal cortex | Subiculum | -0.09 | 0.674 |

**Supplementary Table 17. Details of projection associations between NfL the superficial layers of the entorhinal cortex and synaptic density in interconnected hippocampal subregions.** As this analysis was exploratory, p-values were not corrected for multiple comparisons.

| **Group** | **Projecting region (NfL)** | **Target region (synaptic density)** | **r-value** | **p-value** |
| --- | --- | --- | --- | --- |
| Controls + AD | Superficial layers of entorhinal cortex | CA1 | 0.03 | 0.849 |
|  | Superficial layers of entorhinal cortex | Subiculum | 0.15 | 0.366 |

**Supplementary Table 18. Detailed association model between synaptic density and cognitive scores.** P-values are FDR corrected for multiple subregions. Significant associations are in bold.

| **Group** | **Subregion** | **r-value** | **R^2^** | **95% CI** | **ΔCDR** per 1 SD decrease in synaptic density | **p-value** |
| --- | --- | --- | --- | --- | --- | --- |
| AD | CA4 | -0.38 | 14.2% | [-0.059, 0.006] | -0.022 | 0.143 |
|  | CA2 | -0.40 | 16.2% | [-0.060, 0.004] | -0.023 | 0.110 |
|  | CA1 | **-0.64** | **41.0%** | **[-0.083, -0.021]** | **-0.044** | **0.003** |
|  | Subiculum | **-0.62** | **38.3%** | **[-0.072, -0.015]** | **-0.041** | **0.012** |
|  | Entorhinal cortex | **-0.60** | **35.5%** | **[-0.065, -0.013]** | **-0.039** | **0.008** |
|  | Parahippocampal gyrus | **-0.48** | **22.6%** | **[-0.084, -0.005]** | **-0.037** | **0.041** |
|  | **Fusiform gyrus** | **-0.67** | **45.1%** | **[-0.092, -0.027]** | **-0.049** | **0.002** |

**Legend:** *ΔCDR: predicted change in CDR; AD: Alzheimer’s disease; CA: Cornu Ammonis; CDR: clinical dementia rating; CI: confidence interval; R^2^: determination coefficient*

# References

1. Thal DR. Rub U. Orantes M. Braak H. Phases of A beta-deposition in the human brain and its relevance for the development of AD. Neurology. 2002;58(12):1791-800.

2. Braak H. Alafuzoff I. Arzberger T. Kretzschmar H. Del Tredici K. Staging of Alzheimer disease-associated neurofibrillary pathology using paraffin sections and immunocytochemistry. Acta Neuropathol. 2006;112(4):389-404.

3. Braak H. Del Tredici K. Rub U. de Vos RA. Jansen Steur EN. Braak E. Staging of brain pathology related to sporadic Parkinson's disease. Neurobiol Aging. 2003;24(2):197-211.

4. Montine TJ. Phelps CH. Beach TG. Bigio EH. Cairns NJ. Dickson DW. et al. National Institute on Aging-Alzheimer's Association guidelines for the neuropathologic assessment of Alzheimer's disease: a practical approach. Acta Neuropathol. 2012;123(1):1-11.
